# The Olive Oil Monophenolic Secoiridoid Ligstroside Aglycone Suppresses Melanoma Progression by Targeting the BRAF Signaling Pathway

**DOI:** 10.3390/molecules30010139

**Published:** 2025-01-01

**Authors:** Md Ashiq Mahmud, Abu Bakar Siddique, Afsana Tajmim, Judy Ann King, Khalid A. El Sayed

**Affiliations:** 1 Department of Basic Pharmaceutical and Toxicological Sciences, College of Pharmacy, University of Louisiana at Monroe, 1800 Bienville Drive, Monroe, LA 71201, USA; mahmudma@warhawks.ulm.edu (M.A.M.); siddique.ulm@gmail.com (A.B.S.); afsana.ulm@gmail.com (A.T.); 2 Foundational and Clinical Sciences Department, Thomas F. Frist, Jr. College of Medicine, Belmont University, 1900 Belmont Boulevard, Nashville, TN 37212, USA; judy.king@belmont.edu

**Keywords:** *BRAF V600E* mutation, ELOVL6, GPD1, ligstroside aglycone, melanoma, olive phenolics, tumor necrosis

## Abstract

Melanoma is among the most abundant malignancies in the US and worldwide. Ligstroside aglycone (LA) is a rare extra-virgin olive oil-derived monophenolic secoiridoid with diverse bioactivities. LA dose–response screening at the NCI 60 cancer cells panel identified the high sensitivity of the Malme-3M cell line, which harbors a *BRAF V600E* mutation. Daily oral 10 mg/kg LA exhibited potent in vivo antitumor effects against Malme-3M cells xenograft in a nude mouse model by targeting the BRAF signaling pathway. A human Clariom S microarray analysis of the collected Malme- 3M tumors identified 571 dysregulated genes, with the downregulation of pathways critical for melanoma cells growth and survival. A Western blot analysis of the collected animal tumors further validated the downregulation of the mutated BRAF–MAPK axis, as well as the GPD1 and ELOVL6 expression levels. A histopathological analysis of Malme-3M tumor sections showed extensive focal tumor necrosis in treated mice. An immunofluorescence study of tumor sections showed notable reductions in proliferation marker ki67 and the vasculogenesis marker CD31 in treated tumors. These findings promote LA as a potential nutraceutical lead for the control of the *BRAF V600E* mutant melanoma.

## 1. Introduction

Melanoma is the 5th most common cancer in United States, with an estimated 100,640 new annual cases in 2024, along with 5430 and 2860 prospective deaths among males and females, respectively [1]. Melanoma is a prevalent malignancy in the northern hemisphere, with a dramatic 30-fold increase in incidence rates over the past century among US residents, with individuals of European descent facing three times the risk compared to Asians and fifteen times that of those with South American or African origins [1,2]. The non-melanoma skin cancers like basal cell carcinoma and squamous cell carcinoma are less aggressive, unlike the rare cutaneous melanoma, which is highly lethal and causes 80% of skin cancer deaths with high metastatic risk [3]. Recurrent BRAF or NRAS mutations in melanoma activate the MAPK pathway, triggering activated ERK signaling, leading to an aggressive tumor profile with enhanced cell proliferation and survival. These mutations are pivotal in melanoma’s aggressiveness and resistance to therapies, making them crucial molecular targets for developing new effective interventions [4].

The *BRAF* proto-oncogene, found on chromosome 7′s long arm (7q34), is responsible for encoding a protein that transmits proliferative signals from the cell surface to the nucleus. As a serine/threonine kinase, this protein is an integral part of the MAPK pathway (RAS/RAF/MEK/ERK), which is essential for controlling cell growth, division, differentiation, migration, and survival [5,6]. In melanoma, *BRAF* mutations are early and significant in nearly 50% of cases [7]. The most prevalent mutation is *BRAF V600E*, a valine-to-glutamate substitution in the kinase domain, leading to a constant protein kinase activation, including MEK/ERK signaling cascade, resulting in melanocyte hyperproliferation and contributing to melanoma development [8].

BRAF inhibitors are available for patients with aggressive *BRAF*-mutant melanoma that has progressed following prior immunotherapy, as it enhances response rates and extends both progression-free and overall survival [9,10]. Vemurafenib (Zelboraf^®^) and Dabrafenib (Tafinlar^®^) are available small-molecule inhibitors targeting the *BRAF V600E* mutation, demonstrating significantly improved overall survival rates without similar efficacy against the wild-type melanomas [11,12,13,14]. *BRAF V600E* mutation-targeting drugs usually develop resistance within 6 months of continued use, diminishing their efficacy and increasing the risk of tumor recurrence [12]. Clinical trials have reported several cutaneous, vascular, and systemic adverse effects, suggesting a dire need for better alternatives [15,16,17].

The extra-virgin olive oil (EVOO) phenolics have been compellingly and thoroughly associated with a wide range of positive health benefits, including oncological, neurological and cardiovascular directions [18,19,20,21]. Among these, the EVOO secoiridoids—a subclass of iridoids characterized by the cleavage of their cyclopentane ring—are notable for their bioactivity. The EVOO monophenolic secoiridoid *S*-ligstroside aglycone (LA) has been much less investigated compared to the other EVOO phenolics oleocanthal, oleacein, and oleuropein aglycone, as well as their parent simple alcohols tyrosol and hydroxytyrosol [18,19]. This may be attributed to its scarce natural occurrence in EVOO [20,21]. Oleocanthal significantly inhibited human melanoma cells growth by inhibiting ERK1/2 and AKT phosphorylation, and downregulating Bcl-2 expression [18]. Oleocanthal also suppressed the growth of non-melanoma skin cancer cells by repressing B-Raf expression [19]. An EVOO enriched with LA and oleocanthal demonstrated notable anti-proliferative effects against human liver cancer cell lines, primarily through the induction of autophagy [20]. LA exhibited significant anti-inflammatory properties in LPS-stimulated murine peritoneal macrophages [21]. This anti-inflammatory action was mediated via the modulation of MAPKs, JAK/STAT, NF-κB, Nrf2/HO-1, and the NLRP3 inflammasome [21]. A potent tumor-killing effect associated with elevated levels of apoptotic cell death markers was reported for LA against SKBR3, MCF-7, and HER2-positive breast cancer cell lines, with the downregulation of FAS and HER2 levels [22,23]. LA demonstrated moderate cytotoxic effects against a panel of 16 human cancer cell lines in vitro [24]. LA did not inhibit c-MET activation in a Z′-LYTE™ kinase cell-free assay, but showed optimal antimigratory and anti-invasive activities against the TNBC cells MDA-MB231, with an IC_50_ of 13.8 µM [25]. Recently, LA and its acylated analogs decreased the expression of pro-inflammatory genes NOS2 and MMP13 at both RNA and protein levels [26]. LA also reduced NO release and suppressed proteoglycan loss in human osteoarthritis cartilage [26].

Thus, LA was submitted to the NCI-DTP 60 cell lines panel to screen its potential against rationally selected cancer cell lines (Supplementary Figure S1). LA demonstrated superior in vitro efficacy against various NCI melanoma cell lines and was therefore selected for this study to elucidate its anti-melanoma molecular mechanism. It is worth noting that LA passed the single-dose testing, progressing to the next level, by showing good activity against various lung, ovarian, renal cell, and other cancer cell lines, underscoring its anticancer potential. Exploring LA molecular mechanisms against metastatic melanoma could lead to improved therapeutic alternatives. Thus, this study seeks to investigate the effects of LA in targeting melanoma.

## 2. Results and Discussion

### 2.1. Dose–Response Screening of Ligstroside Aglycone (LA) in the NCI DTP 60 Cancer Cell Line Panel

**Figure 1:**
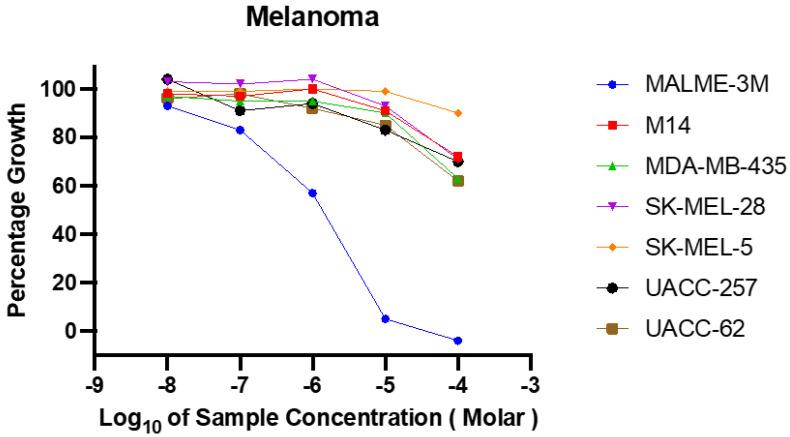
LA showed potent dose–response in vitro anti-melanoma activity against the NCI-DPT melanoma cancer cell line panel.

The classic NCI60 cell line screen panel is composed of nine malignancy types: leukemia, non-small-cell lung, melanoma, colon, CNS, ovarian, renal, prostate, and breast cancers [27]. This standardized panel proved useful to screen and discover small-molecule therapeutic hits with promising anticancer potential. The NCI60 cell lines are well-characterized human cancer cell lines that are extensively genomic and proteomic profiled, including gene mutations, amplifications and deletions, proteomics, the methylome, microRNA, and exosomes [27]. Ligstroside aglycone (LA) was initially screened for antiproliferative activity against the NCI Developmental Therapeutics Program (DTP) classic 60 cancer cell line panel at an initial testing dose of 10 µM protocol (NSC: D-768074) [27]. LA exerted a promising cytotoxicity profile, and therefore it was subjected to the secondary assays, a more detailed five-dose response testing procedure. LA showed significant cytotoxic effects, particularly against the melanoma cell lines (Figure 1). Among the seven melanoma cell lines tested, the Malme-3M cell line exhibited the most pronounced dose-dependent sensitivity (Supplementary Figure S2). This melanoma cell line has a mutation in the *BRAF* gene (p.V600E), a driver mutation known to play a role in the activation of the MAPK signaling pathway in cancer [12,13,14,15,16,17].

Consistent with the results of the one-dose assay, LA demonstrated potent cytotoxicity, with particularly high sensitivity observed against the Malme-3M cell line, which showed over 75% mean cell death (Figure 1). The calculated mean Log_10_ GI_50_ (growth inhibitory power 50) value for LA against the Malme-3M cell line was −5.86 mol/L, the Log_10_ TGI (total growth inhibition) was −4.45 mol/L, and the Log_10_ LC_50_ (lethal concentration 50) was >−4.0 mol/L. The most potent BRAF, MEK, and ERK inhibitors showed a mean GI_50_ value of –6.5 mol/L (0.32 μM), while the least potent inhibitor showed a mean GI_50_ value of –4.35 mol/L (45 μM) [27]. The heightened sensitivity for LA against the Malme-3M cell line is likely due to the *BRAF V600E* mutation, with a wild-type NRAS high occurrence in this cell line, whereas other melanoma lines harboring NRAS mutations may activate alternate pathways like PI3K-Akt to bypass the inhibition of the MAPK signaling pathway [28,29]. The microscopic images of the Malme-3M cell line treated with various LA concentrations showed a clear dose-dependent inhibition of cell growth and proliferation (Supplementary Figure S2). The images reveal a reduction in cell numbers and morphology changes in time and dose-dependent manners, which is consistent with the LA NCI cytotoxicity.

### 2.2. Suppressive In Vivo LA Effects Against Malme-3M Cells in a Nude Mouse Xenograft Model

Following the potent in vitro results against Malme-3M melanoma cells, we further investigated LA in vivo antitumor efficacy using a nude mouse xenograft model. Ten female nude mice were subcutaneously xenografted Malme-3M cells, as detailed in the Materials and Methods section. After nearly 60 days of initial tumor cell xenografting, the mice tumors averaged 50 mm^3^. Mice were then randomly assigned into two groups, namely (i) vehicle control and (ii) LA treatment, with *n* = 5 each. The treatment group were administered LA orally daily using an oral gavage at a dose of 10 mg/kg in 100 μL vehicle control (VC, 0.1% *v*/*v* sterile DMSO in sterile PBS). The other group were administered VC. The treatments continued for an additional 40 days. All mice were sacrificed at the end of the study, and the tumors and organs were collected, weighed, and sectioned.

**Figure 2:**
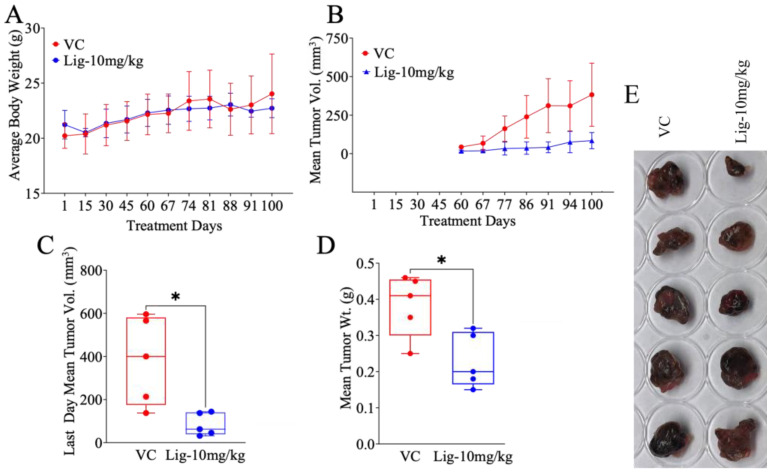
The anti-melanoma effects of LA in a nude mice xenograft model. (**A**) Mice body weight monitoring over the study’s 100-day course, showing no significant body weight difference between treatment versus VC group. (**B**) Tumor volume monitoring over the 40-day dosing course, showing progressive tumor volume reduction in the LA treatment group versus the VC group. (**C**) A comparison of the mean tumor volume reduction in the LA-treated group (blue) versus the VC-treated (red) mouse groups on the last day. (**D**) A mean tumor weight comparison of the LA-treated (blue) versus the VC-treated (red) groups, showing a statistically significant tumor weight reduction in the LA group. (**E**) Representative tumor images at the end of the study, displaying significant tumor suppression in LA-treated group versus VC. Student’s *t*-test was used, where * *p* < 0.05 indicating statistical significance.

The results demonstrated that the LA-treated group had a significant reduction in tumor progression compared to the VC-treated group. The mean collected tumor weight was 230 mg and 384 mg in the LA- and VC-treated groups, respectively (Figure 2A). Daily monitoring of tumor volume over the study course revealed a gradual and consistent reduction in the tumor volume over the treatment period (Figure 2B,C). On the last study day, the mean tumor volume in the control group reached nearly 382.4 mm^3^, while the LA-treated group’s mean tumor volume was reduced to nearly 84.22 mm^3^ (Figure 2C). The notable difference between the tumor weight and volume suppression results is worth noting, which might be justified by the variations in tumor weights due to their variable association with liquids from the microenvironment or possible subjective tumor volume variation during the measuring by the digital caliber. Importantly, LA treatment did not result in any significant decrease in body weight beyond what VC caused, suggesting a possible favorable safety profile (Figure 2D). A morphological inspection of the collected tumors revealed a pronounced tumor size reduction in the LA treatment group versus VC (Figure 2B–E). 

The literature has linked the antitumor effects of LA and EVOO secoiridoids to their ability to disrupt key signaling pathways critical for tumor survival and proliferation. LA is reported to modulate the MAPK and NF-κB pathways, both of which are essential in melanoma pathogenesis and resistance to therapy [21,30]. Given the observed robust LA tumor suppressive effects and prospective safety profile, LA holds promise as a therapeutic nutraceutical for melanoma.

### 2.3. LA Induces Malme-3M Focal Tumor Necrosis

**Figure 3:**
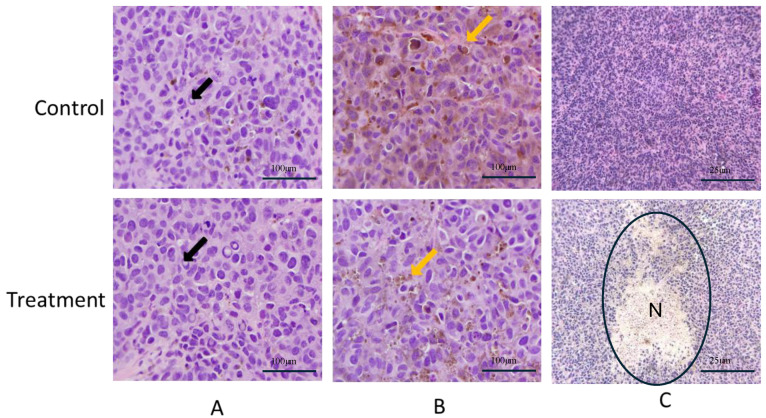
A histopathological study of representative hematoxylin and eosin (H&E)-stained sections of collected Malme-3M tumors from VC- and LA-treated nude mice. (**A**) Images showing the presence of nuclear pseudoinclusions (black arrows) and (**B**) melanin pigments (yellow arrows) in both VC- and LA-treated tumor sections (40× objective, 400×). (**C**) The detection of focal necrosis areas in LA-treated Malme-3M tumors, not found in VC-treated tumor sections. N: Focal necrosis area (10× objective 100×).

An immunohistochemical analysis of collected tumor sections from both VC- and LA-treated mice revealed several common and variation histopathological features. The common features included numerous nuclear pseudoinclusions, consistently observed across both the LA and VC treatment groups, indicative of cellular alterations within the tumor microenvironment (Figure 3A). Additionally, melanin pigment was also detected in tumors from both groups, suggesting the presence of melanocytic elements (Figure 3B).

The most distinct histopathological feature observed in the LA-treated tumor sections included focal areas of necrosis, which were absent in the VC group. This focal necrosis suggests that LA may exert a localized cytotoxic effect within the tumor tissue, contributing to its cells’ death effect (Figure 3C). Focal necrosis in tumors treated with cytotoxic agents is a well-documented phenomenon shown in various anticancer agents, inducing localized tumor tissues necrosis as a part of their cytotoxic effects [31,32]. Focal necrosis can result from insufficient blood supply or increased metabolic stress in tumor regions with high drug concentrations [33]. The identification of focal necrosis in LA-treated tumors suggests that LA may have a dual mode of action: inhibiting tumor cells proliferation and inducing localized cell death, which could enhance its therapeutic potential [33,34,35].

### 2.4. Comparison of Ki67 and CD31 Expression in Malme-3M Tumor Sections Using Immunofluorescence

**Figure 4:**
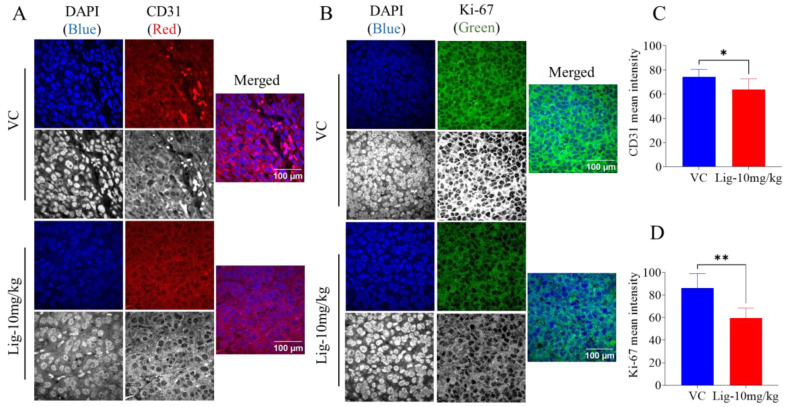
An immunofluorescence analysis of LA effects against ki67 and CD31 in Malme-3M tumors. (**A**,**B**). Images presenting ki67 and CD31 expression in Malme-3M tumors treated with LA versus VC-treated mouse tumors, as detected by immunofluorescence staining and analyzed by confocal microscopy. (**C**,**D**). Vertical bar graphs showing normalized integrated fluorescence intensity values, representing the ki67 and CD31 expression levels in Malme-3M tumors from LA-treated versus VC-treated mice. Immunofluorescence intensity staining was quantified in several optical fields, as detailed in Materials and Methods section. Student’s *t*-test used for statistical analysis. * *p* < 0.05 and ** *p* < 0.01 indicate statistical significance.

Immunofluorescence results showed a notable reduction in the expression levels of the proliferation marker ki67 and the vasculogenesis marker CD31 in LA-treated Malme-3M tumor sections, unlike the VC-treated tumors (Figure 4). This clearly justifies LA’s effective antiproliferative activity and potential for potent angiogenesis suppression in Malme-3M tumors.

### 2.5. Comparison of LA- Versus VC-Treated Malme-3M Tumors’ Microarray-Based Gene Expression Signature and Enrichment Analysis

The Human Gene Chip Clariom™ S array analysis was employed to identify the gene expression signature associated with LA treatment in Malme-3M primary tumor tissues as compared to VC. The Clariom™ S array is a next-generation transcriptome-wide gene-level expression-profiling tool, providing comprehensive coverage of all known, well-annotated genes [36].

**Figure 5:**
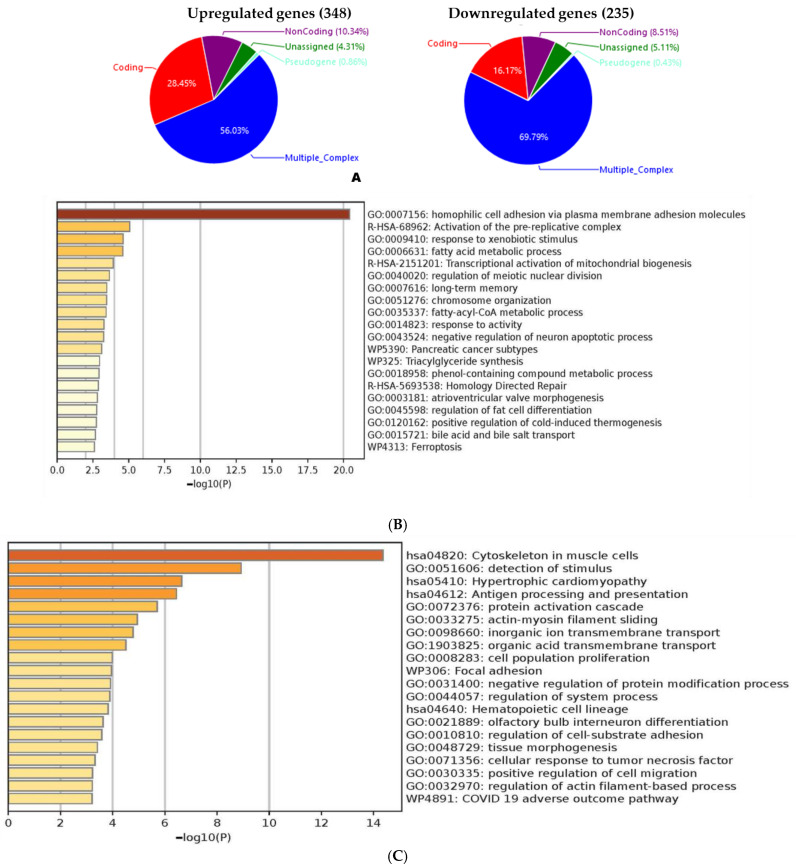
A comparative microarray analysis of vehicle control vs. LA mice tumors using mice and human Clariom S arrays, respectively, highlighting the differential gene expression and ontology (GO) results. (**A**) Mouse tumor array analysis. (**B**) An enrichment analysis of downregulated pathways following LA treatment. LA treatment suppressed key processes such as homophilic cell adhesion, the activation of the pre-replicative complex, carboxylic acid and lipid metabolism, and meiotic nuclear division. (**C**) An enrichment analysis of upregulated pathways following LA treatment. LA upregulated cytoskeleton organization, the detection of stimuli, antigen processing–presentation, lymphocyte proliferation, focal adhesion, and hypertrophic cardiomyopathy pathways.

A comparative analysis of VC- versus LA-treated tumors revealed the significant dysregulation of 583 genes out of 21,448 genes, with a log2 fold change threshold of >2 or <−2 [36]. Of these, 348 genes were upregulated, while 235 genes were downregulated in response to LA treatment. Approximately 56.03% of the upregulated genes were derived from multiple complexes, with 28.45% representing coding genes (Figure 5A, left). For the downregulated genes, nearly 69.79% originated from multiple complexes, while 16.17% were coding genes (Figure 5A, right).

A gene enrichment analysis using Metascape revealed several key biological processes were significantly downregulated following LA treatment [37] (Supplementary Table S1). Notably, homophilic cell adhesion was potentially suppressed via plasma membrane adhesion molecules, which could indicate disrupted cell–cell interactions essential for tumor growth and metastasis. Additionally, the activation of the pre-replicative complex was significantly downregulated, suggesting a potential inhibition of DNA replication initiation, leading to cell cycle arrest and reduced tumor cell proliferation.

Further analysis indicated suppression in pathways related to carboxylic acid and lipid metabolism as well as the fatty-acyl-CoA metabolic process. This downregulation suggests that LA treatment impairs lipid biosynthesis and degradation, thus disrupting the metabolic flexibility required for rapid tumor growth. Additionally, the downregulation of meiotic nuclear division suggests potential broader effects on cell division and genomic stability within the tumor (Figure 5B).

Conversely, LA treatment led to the upregulation of several pathways linked to cellular structure and immune responses (Supplementary Table S2). Cytoskeleton organization in muscle cells and actin–myosin filament sliding were among the most significantly enriched processes, suggesting a possible enhancement of cytoskeletal integrity, which may contribute to limiting tumor cell motility and invasion.

The analysis also revealed upregulation in pathways involved in the detection of stimuli and antigen processing and presentation, suggesting an enhanced immune surveillance and response within the tumor microenvironment. This immune activation could be a contributing factor to the antitumor effects of LA, facilitating the recognition and elimination of tumor cells by the immune system.

Pathways related to lymphocyte proliferation and focal adhesion were upregulated, further indicating a reinforcement of the immune response and cell–matrix interactions, potentially limiting tumor progression. Interestingly, the enrichment of the hypertrophic cardiomyopathy pathway, typically associated with heart disease, suggests that some cytoskeletal changes induced by LA may overlap with those observed in hypertrophic conditions, possibly impacting the mechanical properties of tumor cells (Figure 5C).

These findings suggest that LA exerts its anti-melanoma effects by modulating the expression of key genes involved in critical metabolic and biological processes. The downregulation of pathways and markers essential for tumor cell survival and proliferation, coupled with the upregulation of pathways enhancing cytoskeletal integrity and immune responses, highlights the multifaceted impact of LA on melanoma pathogenesis and suggests high therapeutic potential. A detailed list of the affected 583 genes evidenced by the microarray analysis is given in Supplementary Table S3.

Additionally, gene expression analysis using the bioinformatics platform UALCAN revealed that both GPD1 and ELOVL6 are significantly upregulated in primary versus metastatic skin cutaneous melanoma samples from the TCGA database [38]. The downregulation of these genes in response to LA treatment suggests that it might possess anti-metastatic potential, as these genes are linked to processes critical for tumor progression and angiogenesis, which was further supported by the effective downregulation of ki67 and CD31 [34,35] (Figure 4, Supplementary Figure S3).

To further validate the microarray results, we focused on the two most downregulated genes, glycerol-3-phosphate dehydrogenase 1 (GPD1) and elongation of very long-chain fatty acid 6 (ELOVL6), for further protein expression analysis. GPD1 (cytosolic GPDH) is usually localized at the mitochondria outer membrane facing cytosol. Its function is to catalyze dihydroxyacetone phosphate reduction into glycerol-3-phosphate unlike GPD2 (the mitochondrial GPDH), which is normally embedded on the outer surface of the inner mitochondrial membrane, overlooking the cytosol, catalyzing glycerol-3-phosphate oxidation to dihydroxyacetone phosphate [39,40]. GPD1 and GPD2 both act as a NADH shuttle for mitochondrial bioenergetics and function, linking the glucose and lipid metabolism [40]. GPD1 proved critical for regulating the Embden–Meyerhof glucose glycolysis pathway (E-M pathway), mostly dysregulated in Huntington’s disease and validated as a specific biomarker for dormant glioma stem cells [39,40]. GPD1 is documented as expressed in brain tumor stem cells (BTSCs) but not expressed in normal neural stem cells [39]. The inhibition of GPD1 was hypothesized to suppress BTSC maintenance and therefore extending glioma patients survival and preventing disease relapse [39].

The ELOVL fatty acid elongase 6 (ELVOL6) is a critical enzyme for the fatty acid elongation and can be involved in fat loss in cancer-associated cachexia (CAC) [41]. CAC is originated from malignancies and usually causes debilitating wasting syndrome [41]. ELOVL6 proposed as a biomarker for the early diagnosis of CAC and a potential molecular target. Meanwhile, ELVOL6 loss significantly suppressed the acute myeloid leukemia (AML) propagation, and therefore targeting ELOVL6 activity or pathways regulated by ELOVL6 is considered a novel valid future AML therapeutic strategy [42]. ELOVL6 proved highly overexpressed in colorectal cancer patient tissues’ cultured cell lines like HT-29 and WiDr [43]. The silencing of ELOVL6 reduced colorectal cancer cell proliferation and migration, and was therefore proposed as novel viable molecular target [43].

**Figure 6:**
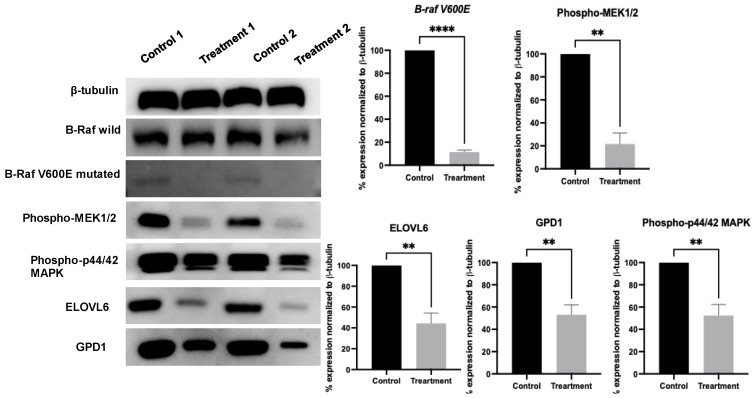
A Western blot Analysis of tumor lysates from control and ligstroside aglycone (LA)-treated groups: expression levels of BRAF wild-type (BRAF WT), phosphorylated MEK (p-MEK), phosphorylated MAPK (p-MAPK), glycerol-3-phosphate dehydrogenase 1 (GPD1), and elongation of very long-chain fatty acid 6 (ELOVL6). β-tubulin was used as the loading control. Densitometric values were normalized to β-tubulin. Student’s *t*-test was used, where ** *p* < 0.01, and **** *p* < 0.0001 indicate statistical significance. Vertical bar graphs indicate the normalized integrated optical density for bands visualized in each lane.

A Western blot analysis of the Malme-3M tumor lysates from mice treated daily with 10 mg/kg of LA showed a notable reduction in both GPD1 and ELOVL6 protein level expression in the treatment group compared to the VC group (Figure 6). LA treatment reduced the expression levels of GPD1 and ELOL6 by 46.3% and 55%, respectively (Figure 6). This result is consistent with the microarray data, supporting the conclusion that LA treatment downregulates these key metabolic genes in Malme-3M collected tumors. This is the first report for the GPD1 and ELOVL6’s critical contributions in melanomas. A recent study demonstrated that dabrafenib-induced metabolite changes occur shortly after treatment initiation, preceding the tumor volume reduction. Tumor concentrations of lactate, alanine, and bioenergetics-related metabolites such as nucleoside triphosphates (NTP) and inorganic phosphate correlated with diverse therapeutic responses to dabrafenib [44]. Similarly, GPD1 and ELOVL6 might serve as early biomarkers of therapeutic response to various melanoma interventions, but further validation is needed.

### 2.6. Modulation of BRAF Signaling by LA Treatment

The BRAF is a key effector kinase acting downstream of RAS, leading to the oncogenic activation of MEK and ERK [45]. BRAF alterations include the gain-of-function mutations, copy-number alterations, and structural rearrangements. Specifically, *BRAFV600* variants in melanoma are prevalent in 50% of clinical cases [46]. To evaluate the effect of LA on BRAF signaling, a comparative Western blot analysis was performed, comparing VC- versus LA-treated tumor lysate groups. This analysis revealed the consistent expression of *BRAF* wild-type in both the VC- and LA-treated samples, suggesting that LA does not significantly impact the wild-type *BRAF* expression in Malme-3M melanoma cells. In contrast, densitometric analysis indicated the downregulation of the expression of *BRAF V600E* by more than 50% in the LA-treated group compared to VC (Figure 6), indicating a selective effect on the mutant BRAF protein variant.

Additionally, the levels of phosphorylated (activated) MEK (p-MEK) and MAPK (p-MAPK) were both significantly reduced in the LA-treated samples, as confirmed by densitometry. LA reduced the expression levels of mutant *BRAF* by 87.5%, p-MEK by 77.5%, and p-MAPK by 47.5% (Figure 6). The downregulation of p-MEK and p-MAPK aligns with the observed inhibition of *BRAF V600E*, with both proteins showing over a 50% reduction compared to VC (Figure 6). These signaling proteins are critical downstream effectors for the BRAF pathway, and their decreased expression was justified by the effective disruption of the mutant variant *BRAF V600E*-driven signaling cascade. These results are consistent with the literature findings, which validated the *BRAF V600E*-MEK-MAPK axis as a key tumorigenesis driver in melanoma and other cancers harboring this mutation [47,48].

The results of this study validate the hypothesis that LA selectively targets the oncogenic BRAF signaling pathway, sparing the wild-type proteins while suppressing the V600E mutant variant. This selective inhibition and the subsequent reduction in p-MEK and p-MAPK levels align with the known mechanisms by which BRAF inhibitors attenuate tumor progression [49,50]. Furthermore, the downregulation of the p-MEK and p-MAPK highlights the effectiveness of LA’s ability to suppress cell proliferation and survival pathways. The findings of this study may also highlight LA’s therapeutic potential against other malignancies harboring the *BRAF V600E* mutation variant. The results also promote LA as a prospective nutraceutical lead for use in controlling *BRAF V600E* malignancies.

## 3. Materials and Methods

### 3.1. In Vitro Antiproliferative Activity Evaluation Using NCI One-Dose and Five-Dose Assays

LA (NSC: D-768074) was accepted for screening using the classic NCI60 cell line screen panel at the Developmental Therapeutics Program (DTP)—National Cancer Institute (NCI), Bethesda, USA, for evaluation of its antiproliferative activity [27,51,52]. Initially, LA was tested in a one-dose assay at a concentration of 10 µM (dissolved in DMSO) against a panel of 60 cancer cell lines, including leukemia, non-small-cell lung, colon, CNS, melanoma, ovarian, renal, prostate, and breast cancer [27,51,52]. The compound was added to 96-well microtiter plates, incubated at 37 °C for 48 h, and the antiproliferative effect was determined using sulforhodamine B (SRB), a protein-binding dye [27,51,52]. The percentage growth inhibition of the treated cells was compared to the untreated controls, and the results for each cell line were reported based on the 10 µM concentration. Following the one-dose assay, serial 5-fold dilutions of the initial DMSO stock solution were prepared to assess LA’s activity at various concentrations. The compound was further evaluated by the DTP-NCI five-dose assays to determine the dose–response parameters (GI_50_, TGI, and LC_50_) for each cell line. A dose–response curve was established using concentrations of 0.01, 0.1, 1, 10, and 100 µM [27,51,52].

### 3.2. Chemicals, Reagents and Antibodies

LA was isolated from EVOO (The Governor, Corfu, Greece, fall 2020 collection). The extraction and purification followed our previously published method using the liquid–liquid (water–EVOO) extraction, resin entrapment, and final purification of Sephadex LH20 to reach >95% purity level, guided by q^1^HNMR and high-resolution mass analyses [53]. Spectroscopic identity was based on extensive 1D and 2D NMR analyses and a comparison with literature values, especially the chemical shift of protons H-3 (δ 7.59, singlet) and H-8 (δ 9.57, broad singlet) chemical shifts [53,54]. Chemical reagents and solvents were procured from VWR International (Suwanee, GA, USA), unless otherwise specified. Cells and culture reagents were sourced from ATCC (Manassas, VA, USA), and antibodies were purchased from Cell Signaling Technology (Beverly, MA, USA), typically used at a 1:1000 dilution unless otherwise noted.

### 3.3. Cell Lines and Culture Conditions

The human Malme-3M malignant melanoma cell line was obtained from the Charles River Laboratories in Frederick, MD, USA. The cells were cultured in Roswell Park Memorial Institute (RPMI-1640) medium supplemented with 10% *v*/*v* fetal bovine serum (FBS), 2.5 µg/mL amphotericin B, 100 µg/mL streptomycin, and 100 IU/mL penicillin G, and were maintained in a humidified incubator at 37 °C with 5% CO_2_. For sub-culturing, the cells were washed with calcium- and magnesium-free phosphate-buffered saline (PBS) and then treated with 0.05% *v*/*v* trypsin containing 0.02% *v*/*v* ethylenediamine-tetraacetic acid (EDTA) in PBS for 5 min at 37 °C [55].

### 3.4. Compound Preparation and Stock Solution

A 25 mM stock solution was made by dissolving LA in sterile dimethyl sulfoxide (DMSO). This stock solution was subsequently diluted to prepare various concentrations of LA treatments for cell culture experiments. Across all experimental groups, the final concentration of DMSO in the media was consistently maintained below 0.1% *v/v* to ensure minimal solvent interference [55].

### 3.5. Cell Viability Assay

Cells were seeded in 96-well plates at a density of 5 × 10^3^ cells/well (6 replicates/group) in 10% (*v*/*v*) FBS RPMI-1640 media and left to attach overnight. Once the cells were confluent enough in 96 wells, the wells were assigned different treatment groups based on the treatment concentrations (1–100 μM LA, 2-fold dilution) and a control group for comparison (DMSO-PBS only). Subsequently, the 3-(4,5-dimethylthiazol-2-yl)-2,5-diphenyl-tetrazolium bromide (MTT) assay was used to determine IC_50_ (50% cell death) according to the previously described protocols [55]. Optical density was measured at 570 nm using a microplate reader (BioTek, Winooski, VT, USA). A Motic’s AE2000 inverted microscope with 10X objective was used to visualize the cellular viability patterns.

### 3.6. Microarray Gene Expression Profiling

Total RNA was extracted from primary tumor tissues of Malme-3M cells treated with LA or vehicle control (VC) using the TRIzol-Phase Lock Gel protocol [36,56]. RNA quality was assessed with an Agilent TapeStation 4200 RNA ScreenTape assay kit (Agilent, Cat # 5067–5576), which provided an overview of RNA integrity and quantity [36,56]. Further RNA samples were biotin-labeled and hybridized using the human Clariom S array/WT plus assay kit (Life Technologies, Cat # 902926) for comprehensive transcriptomic profiling. All array experiments were performed and the data managed and stored at the University of Kansas Medical Center Genomics Core Facility (Kansas City, KS, USA) [36,56].

### 3.7. Gene Enrichment Analysis

Differentially expressed genes were identified by comparing LA-treated versus vehicle control tumor samples, using a log_2_ fold change threshold of ≥2 or ≤−2, and a *p*-value of ≤0.05. The resulting list of significantly downregulated genes was subjected to gene enrichment analysis using Metascape to identify enriched biological processes, molecular functions, and pathways [37,57]. Enrichment clusters were generated, and the top 20 clusters with their representative enriched terms were identified based on the number of genes involved, the percentage of genes from the input list, and the statistical significance (Log10(P) and Log10(q)) [37,57].

### 3.8. Validation of Downregulated Genes by UALCAN

To further validate the microarray results, the expression levels of the two most significantly downregulated genes GPD1 and ELOVL6 were analyzed using the UALCAN bioinformatics portal [38]. UALCAN is an interactive web tool that allows for gene expression analyses of The Cancer Genome Atlas (TCGA) datasets, including comparisons between primary tumor and metastatic samples. GPD1 and ELOVL6 expression were examined in skin cutaneous melanoma (SKCM) samples to assess their relevance in melanoma progression [58].

### 3.9. Western Blot Analysis

Collected tumor samples were washed with cold Dulbecco’s phosphate-buffered saline (DPBS) [55,56]. A solution of 1 mL DPBS with 10 μL protease inhibitor was prepared. DPBS (1 mg sample/5 μL DPBS) was added to the weighed samples, which were then homogenized using an ultrasonic homogenizer (Qsonica Sonicator, Newtown, CT, USA). Each homogenized sample was resuspended and lysed in radioimmunoprecipitation assay (RIPA) buffer (Qiagen Sciences Inc., Valencia, CA, USA) at 4 °C for 30 min. The lysates were then centrifuged at 14,000× *g* for 10 min, and the supernatants were stored at −80 °C.

Protein concentrations were determined using the Pierce BCA Protein Assay (Bio-Rad, Hercules, CA, USA). Each well of a 10% sodium dodecyl sulfate polyacrylamide gel electrophoresis (SDS-PAGE) gel was loaded with 15 μg of tumor lysate. Proteins were then transferred to polyvinylidene difluoride (PVDF) membranes. The membranes were blocked with 5% *v*/*v* bovine serum albumin (BSA) in 10 mM Tris–HCl containing 50 mM NaCl and 0.1% *v*/*v* Tween 20, pH 7.4 (TBST) for 2 h at room temperature [55,56].

Primary antibodies were incubated with the membranes in wash buffer overnight at 4 °C. The following day, the membranes were washed five times with TBST and incubated with horseradish peroxidase (HRP)-conjugated secondary antibodies against each primary antibody for 1 h at room temperature. The membranes were then washed again five times with TBST [55,56].

Proteins were detected using the ChemiDoc XRS chemiluminescent gel imaging system and analyzed using Image Lab software v6.0.1 (Bio-Rad, Hercules, CA, USA). All experiments were performed at least in triplicates, and representative images are presented in the figures. β-Tubulin was used as a housekeeping protein to ensure equal sample loading in all lanes.

### 3.10. Animal Model and Treatment Mode

The athymic nude mice (Foxn1^nu^/Foxn1^+^, 5–6 weeks old) were purchased from Envigo (Indianapolis, IN, USA) and transported to the animal facility at the University of Louisiana at Monroe [55,56]. Upon arrival, the mice underwent a period of acclimatization for 1 week. During acclimatization, they were housed in sterile filter-top cages containing autoclaved bedding and provided with sterile water. The animal facility maintained controlled environmental conditions, including a temperature of 24 ± 2 °C, relative humidity between 50% and 60%, and a 12 h light–dark cycle. The cages were cleaned, and bedding changed twice a week to ensure hygiene and minimize stress to the animals. All experimental procedures were conducted in compliance with the NIH guidelines and approved by the Institutional Animal Care and Use Committee (IACUC) of the University of Louisiana at Monroe, 24-AUG-KES-01. Animal welfare and experimental protocols were strictly followed to minimize discomfort and distress to the mice throughout the study period.

Malme-3M cells were harvested and counted using a hemocytometer. Approximately 2 × 10^6^ cells in 100 µL of Matrigel were inoculated into the left back flank of the animals [55,56]. Tumor development was monitored daily until palpable tumors appeared at the injection site. When tumors reached a volume of 50 mm^3^, the animals were randomized into two groups: a vehicle control group and an LA-treated group (*n* = 5 per group). At the end of the study, the animals were anesthetized with an intraperitoneal injection of a ketamine/xylazine combination (100 mg/kg and 15 mg/kg, respectively) and then euthanized. Tumors were surgically excised from both groups, with dimensions measured every two days using a digital caliper. Tumor volume was calculated using the formula: tumor volume (mm^3^) = [(length × width^2^)/2]. The excised tumors and organs were weighed and stored at −80 °C for subsequent total protein extraction, Western blot analysis, and microarray analysis [55,56].

### 3.11. Immunofluorescence (IFC) Study

The slides for the IFC study were made from Malme-3M tissues embedded in paraffin, which were sectioned to 5 µm thickness sections by AML Laboratories (Jacksonville, FL, USA). The blocks underwent de-paraffinization in xylene and graded ethanol, with the sections heated in citrate buffer (10 mM sodium citrate, pH 6) for 20 min and then permeabilized in TBST solution for 15 min at 25 °C [55,56]. The sections were then stained with the primary antibodies of ki67 (Cat #9129, 1:200, Cell Signaling, Boston, MA, USA) or CD-31 (Cat #3528, 1:200, Cell Signaling) and further diluted in blocking solution for 24 h at 4 °C [55,56]. On the following day, the sections were washed, having been stained with the secondary antibodies for 1 h prior. At the end of the experiment, slides were mounted. All images were captured at the Research Core Facility, LSUHSC, Shreveport, LA, USA, using 100× magnification (Olympus UPLANAPO 100×, 1.50NA) on an Olympus iXplore CSU W1 spinning disk confocal microscope (Center Valley, PA, USA) [55]. Fluorescence intensity (FI) for ki67 and CD31 was quantified using Fiji software v2.9.0 (ImageJ-win64, NIH, USA) [59]. The mean brightness values were measured within the whole regions of the captured multiple images, with background signals subtracted. The mean intensity of ki67 and CD31 were calculated as markers to assess tumor cell proliferation and vascularization, respectively, in VC- and LA-treated groups.

### 3.12. Haematoxylin and Eosin (H&E) Staining

Tumors were promptly harvested and fixed in 10% *v*/*v* neutral buffered formalin for 48 h. After fixation, they were transferred to 70% *v*/*v* ethanol, processed, and embedded in paraffin. The sectioning and hematoxylin and eosin Y staining procedures were carried out at AML Laboratories (Jacksonville, FL, USA), following established protocols [55,56].

### 3.13. Statistical Analysis

Statistical analyses were performed using GraphPad Prism software (versions 8 and 9.3.1, La Jolla, CA, USA). The results were expressed as the mean ± standard deviation (SD) from at least three independent experiments. Differences between two groups (control vs. treatment) were assessed using Student’s two-tailed *t*-test, while differences among three or more groups (control vs. various treatment concentrations) were evaluated using Student’s *t*-test. Statistical significance was defined as * *p* < 0.05, ** *p* < 0.01, *** *p* < 0.001, and **** *p* < 0.0001 when compared to the vehicle-treated control group.

## 4. Conclusions

This study highlights the EVOO-derived monophenolic secoiridoid LA’s potential as an effective anti-melanoma lead in a melanoma xenograft model, with a comprehensive tracing of its molecular targets. LA interfered with crucial melanoma cell survival processes by inhibiting the BRAF–MAPK signaling pathway. This disruption resulted in significant tumor focal necrosis. The downregulation of GPD1 and ELOVL6 further highlighted LA’s suppressive impact on melanoma metabolic pathways critical for tumor progression and survival. The observed sensitivity of *BRAF V600E*-mutated melanoma cells to LA, particularly in the Malme-3M cell line, underscored its potential therapeutic applicability. The molecular and mechanistic link between GPD1-ELOVL6 with *BRAF V600E* in melanoma is yet to be established. The findings of this study suggest that LA could serve as a prospective future nutraceutical candidate for controlling melanoma and other malignancies with *BRAF* mutations.

## Data Availability

The data used to support the findings of this study can be made available by the corresponding author upon request.

## Supplementary Materials

The following supporting information can be downloaded at: https://www.mdpi.com/article/10.3390/molecules30010139/s1, Supplementary Figure S1: LA Developmental Therapeutics Program one-dose mean graph; Supplementary Figure S2: Effect of LA treatments on Malme-3M cell viability. Supplementary Figure S3: The clinical relevance of GPD1 and ELOVL6 expression in subgroups of skin cutaneous melanoma (SKCM); Supplementary Table S1: The top 20 clusters with their corresponding enriched terms for downregulated genes; Supplementary Table S2: The top 20 clusters with their corresponding enriched terms for upregulated genes; Supplementary Table S3: Detailed microarray data for molecular effects of LA on Malme-3M collected tumors.
