# Supplementary material for: The Olive Oil Monophenolic Secoiridoid Ligstroside Aglycone Suppresses Melanoma Progression by Targeting the BRAF Signaling Pathway
Source: Molecules. 2025 Jan 1;30(1):139. doi: 10.3390/molecules30010139 (PMC11721798; doi:10.3390/molecules30010139)
Supplement: Supplementary file 1 [file molecules-30-00139-s001.zip › molecules-3318714-supplementary.pdf]

molecules

MDPI

Article Supplementary Files

The Olive Oil Monophenolic Secoiridoid Ligstroside Aglycone Suppresses Melanoma Progression by Targeting the BRAF Signaling Pathway

Md Ashiq Mahmud 1,†, Abu Bakar Siddique 1,†, Afsana Tajmim 1, Judy Ann King 2 and Khalid A. El Sayed 1,*

1 Department of Basic Pharmaceutical and Toxicological Sciences, College of Pharmacy, University of Louisiana at Monroe, 1800 Bienville Drive, Monroe, LA 71201, USA; mahmudma@warhawks.ulm.edu (M.A.M.); siddique.ulm@gmail.com (A.B.S.); afsana.ulm@gmail.com (A.T.)
2 Foundational and Clinical Sciences Department, Thomas F. Frist, Jr. College of Medicine, Belmont University, 1900 Belmont Boulevard, Nashville, TN 37212, USA; judy.king@belmont.edu
* Correspondence: elsayed@ulm.edu; Tel.: +1-318-342-1725
† These authors contributed equally to this work.

Supplementary Figure S1. LA Developmental Therapeutics Program One Dose Mean Graph.

**NSC:** D-768074 / 1	**Conc:** 1.00E-5 Molar	**Test Date:** Nov 05, 2012
**Experiment ID:** 1211OS74		**Rep rt Date:** Mar 20, 2013

Panel/Cell Line	Growth Percent
Leukemia	
CCRF-CEM	40.62
HL-60(TB)	41.25
K-562	38.47
MOLT-4	84.71
RPMI-8226	7.85
SR	76.23
Non-Small Cell Lung Cancer	
A549/ATCC	61.46
HOP-62	68.23
HOP-92	16.23
NCI-H226	88.90
NCI-H23	73.88
NCI-H322M	85.75
NCI-H460	73.96
NCI-H522	48.91
Colon Cancer	
COLO 205	71.40
HCC-2998	85.12
HCT-116	48.73
HCT-15	97.89
HT29	51.60
KM12	53.24
SW-620	55.95
CNS Cancer	
SF-268	75.86
SF-295	32.70
SF-539	97.08
SNB-19	73.07
SNB-75	75.78
U251	37.04
Melanoma	
LOX IMVI	68.73
MALME-3M	-13.91
M14	39.32
MDA-MB-435	58.99
SK-MEL-2	65.62
SK-MEL-28	51.63
SK-MEL-5	41.56
UACC-257	25.21
UACC-62	38.91
Ovarian Cancer	
IGROV1	66.98
OVCAR-3	19.68
OVCAR-4	56.63
OVCAR-5	91.79
OVCAR-8	43.85
NCI/ADR-RES	101.67
SK-OV-3	91.00
Renal Cancer	
786-0	83.01
A498	133.49
ACHN	34.75
CAKI-1	90.91
RXF 393	88.94
SN12C	58.01
TK-10	63.80
UO-31	55.51
Prostate Cancer	
PC-3	29.83
DU-145	86.92
Breast Cancer	
MCF7	51.85
MDA-MB-231/ATCC	88.38
HS 578T	70.46
BT-549	81.05
T-47D	37.57
MDA-MB-468	29.69
Mean	60.91
Delta	74.82
Range	147.40

Mean Growth Percent - Growth Percent

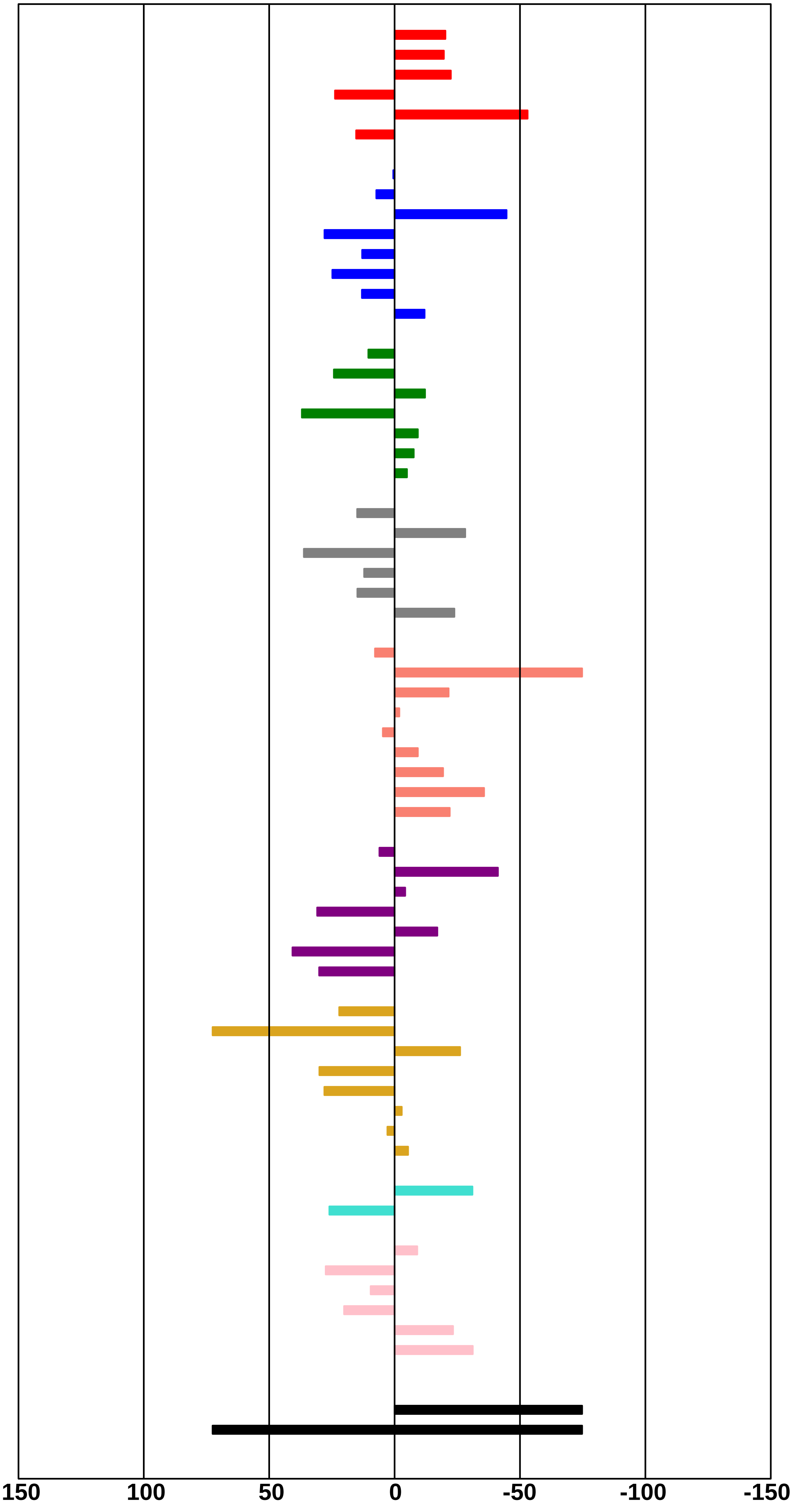

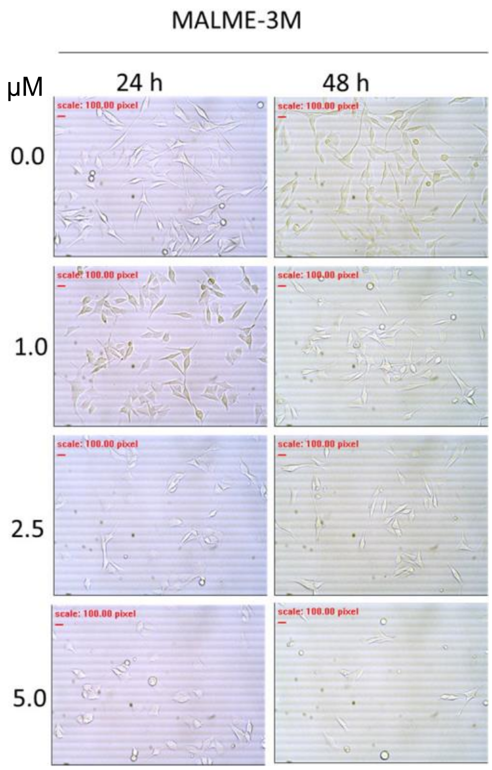

Supplementary Figure S2. Effects of LA treatments on the in vitro viability of Malme-3M melanoma cells. Microscopic images (10X) of Malme-3M cells treated with LA at varying concentrations. Scale bar: 100 pixels/unit.

Supplementary Table S1. The top 20 clusters with their corresponding enriched terms for downregulated genes following LA treatment are presented, with one representative term per cluster. "Count" refers to the number of genes from the user-provided list associated with a specific ontology term. "%" indicates the proportion of all user-provided genes that are linked to that particular ontology term (calculated only from input genes with at least one ontology term annotation). "Log10(P)" represents the p-value in logarithmic base 10, reflecting the statistical significance of the enrichment. "Log10(q)" is the adjusted *p*-value in log base 10, accounting for multiple testing to provide a more precise significance assessment.

GO	Category	Description	Count	%	Log10(P)	Log10(q)
GO:0007156	GO Biological Processes	homophilic cell adhesion via plasma membrane adhesion molecules	23	8.71	-20.26	-15.91
R-HSA-68962	Reactome Gene Sets	Activation of the pre-replicative complex	5	1.89	-5.03	-1.28
GO:0019752	GO Biological Processes	carboxylic acid metabolic process	20	7.58	-4.64	-1.11
GO:0009410	GO Biological Processes	response to xenobiotic stimulus	14	5.30	-4.57	-1.11
R-HSA-556833	Reactome Gene Sets	Metabolism of lipids	19	7.20	-4.38	-1.04
WP5233	WikiPathways	Arsenic metabolism and reactive oxygen species generation	3	1.14	-3.99	-0.79
R-HSA-2151201	Reactome Gene Sets	Transcriptional activation of mitochondrial biogenesis	5	1.89	-3.89	-0.78
GO:0040020	GO Biological Processes	regulation of meiotic nuclear division	4	1.52	-3.57	-0.59
GO:0007616	GO Biological Processes	long-term memory	4	1.52	-3.44	-0.56
GO:0035337	GO Biological Processes	fatty-acyl-CoA metabolic process	4	1.52	-3.39	-0.53
GO:0043524	GO Biological Processes	negative regulation of neuron apoptotic process	7	2.65	-3.24	-0.42

GO	Category	Description	Count	%	Log10(P)	Log10(q)
WP5390	WikiPathways	Pancreatic cancer subtypes	4	1.52	-3.09	-0.33
WP325	WikiPathways	Triacylglyceride synthesis	3	1.14	-2.94	-0.22
GO:0007129	GO Biological Processes	homologous chromosome pairing at meiosis	4	1.52	-2.93	-0.22
WP2355	WikiPathways	Corticotropin releasing hormone signaling pathway	5	1.89	-2.87	-0.19
GO:0003181	GO Biological Processes	atrioventricular valve morphogenesis	3	1.14	-2.83	-0.19
R-HSA-110373	Reactome Gene Sets	Resolution of AP sites via the multiple-nucleotide patch replacement pathway	3	1.14	-2.83	-0.19
GO:0045598	GO Biological Processes	regulation of fat cell differentiation	6	2.27	-2.74	-0.14
GO:0120162	GO Biological Processes	positive regulation of cold-induced thermogenesis	5	1.89	-2.71	-0.13
GO:0015721	GO Biological Processes	bile acid and bile salt transport	3	1.14	-2.65	-0.10

Supplementary Table S2. The top 20 clusters with their corresponding enriched terms for upregulated genes following LA treatment are presented, with one representative term per cluster. "Count" refers to the number of genes from the user-provided list associated with a specific ontology term. "%" indicates the proportion of all user-provided genes that are linked to that particular ontology term (calculated only from input genes with at least one ontology term annotation). "Log10(P)" represents the *p*-value in logarithmic base 10, reflecting the statistical significance of the enrichment. "Log10(q)" is the adjusted *p*-value in log base 10, accounting for multiple testing to provide a more precise significance assessment.

GO	Category	Description	Count	%	Log10(P)	Log10(q)
hsa04820	KEGG Pathway	Cytoskeleton in muscle cells	23	6.46	-14.28	-9.93
GO:0051606	GO Biological Processes	detection of stimulus	29	8.15	-8.72	-5.08
hsa05410	KEGG Pathway	Hypertrophic cardiomyopathy	10	2.81	-6.56	-3.33
hsa04612	KEGG Pathway	Antigen processing and presentation	8	2.25	-5.41	-2.41
GO:0055001	GO Biological Processes	muscle cell development	11	3.09	-5.11	-2.14
GO:0033275	GO Biological Processes	actin-myosin filament sliding	4	1.12	-4.91	-1.96
GO:0098660	GO Biological Processes	inorganic ion transmembrane transport	23	6.46	-4.71	-1.79
GO:0072378	GO Biological Processes	blood coagulation, fibrin clot formation	4	1.12	-4.51	-1.65
GO:1905039	GO Biological Processes	carboxylic acid transmembrane transport	9	2.53	-4.29	-1.46
GO:2001224	GO Biological Processes	positive regulation of neuron migration	4	1.12	-4.20	-1.41
GO:0021537	GO Biological Processes	telencephalon development	12	3.37	-3.95	-1.23
GO:0046651	GO Biological Processes	lymphocyte proliferation	8	2.25	-3.90	-1.20
WP306	WikiPathways	Focal adhesion	10	2.81	-3.88	-1.20
GO:0044057	GO Biological Processes	regulation of system process	18	5.06	-3.87	-1.20

GO:0048729	GO Biological Processes	tissue morphogenesis	18	5.06	-3.77	-1.15
hsa04640	KEGG Pathway	Hematopoietic cell lineage	7	1.97	-3.76	-1.15
GO:0045936	GO Biological Processes	negative regulation of phosphate metabolic process	14	3.93	-3.76	-1.15
GO:0030335	GO Biological Processes	positive regulation of cell migration	18	5.06	-3.70	-1.13
GO:0021889	GO Biological Processes	olfactory bulb interneuron differentiation	3	0.84	-3.61	-1.07
GO:0010810	GO Biological Processes	regulation of cell-substrate adhesion	10	2.81	-3.51	-0.98

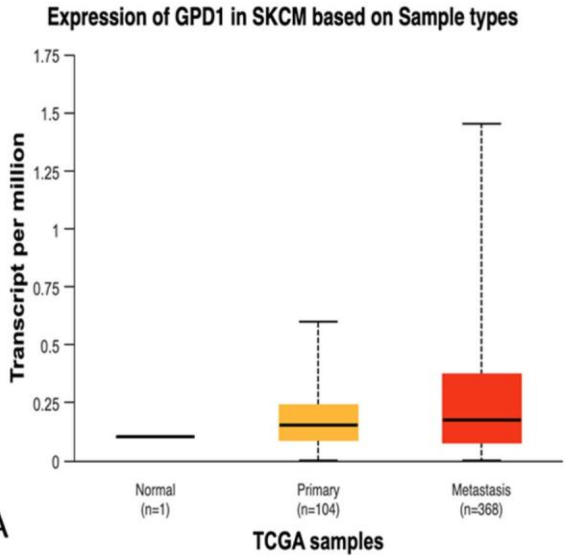

A

Comparison	Statistical significance
Normal-vs-Primary	N/A
Normal-vs-Metastasis	N/A
Primary-vs-Metastasis	1.705380E-01

B

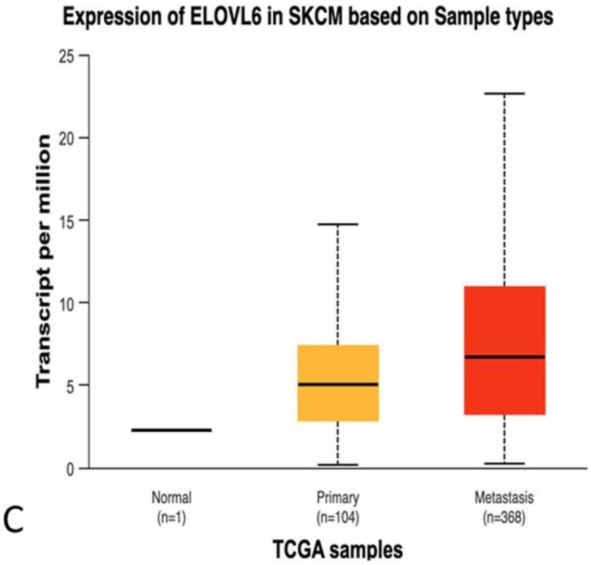

C

Comparison	Statistical significance
Normal-vs-Primary	N/A
Normal-vs-Metastasis	N/A
Primary-vs-Metastasis	3.71960000000726E-05

D

Supplementary Figure S3. Clinical relevance of GPD1 and ELOVL6. Box-and-whisker plots showing the expression of GPD1 and ELOVL6 in subgroups of skin cutaneous melanoma (SKCM) patients. (A) Box plot showing the relative expression of GPD1 in normal, primary, and metastatic SKCM patient samples. (B) Statistical comparison of significance levels for GPD1 expression. (C) Box plot showing the relative expression of ELOVL6 in normal, primary, and metastatic SKCM patient samples. (D) Statistical comparison of significance levels for ELOVL6 expression.

Supplementary Table S3. Microarray data for LA treatment effect of Malme 3M tumors.

#%result_name=Analysis_17 MALME
#%array_type=Clariom_S_Human
#%annotation=Clariom_S_Human.r1.na36.hg38.a1.transcript.csv
#%comparison=treatment vs control

ID	treatment Avg (log	control Avg (log2)	Fold Change	Gene Symbol	Description	Group
TC1200007	4.58	7.4	-7.05	GPD1	glycerol-3-phosphate dehydrogenase 1	Multiple_Complex
TC0400012	8.53	11.3	-6.81	ELOVL6	ELOVL fatty acid elongase 6	Multiple_Complex
TC0100009	5.75	8.21	-5.47	PLPPR4	phospholipid phosphatase related 4	Multiple_Complex
TC0700008	8.64	10.73	-4.27	PRKAR2B	protein kinase, cAMP-dependent, regulatory, type II, beta	Multiple_Complex
TSUnmapp	4.1	6.18	-4.22	KIF15	kinesin family member 15	Coding
TC0500011	4.85	6.85	-4	CDO1	cysteine dioxygenase type 1	Multiple_Complex
TC1000009	2.9	4.8	-3.74	CCDC172	coiled-coil domain containing 172	Multiple_Complex
TC1200007	6	7.9	-3.73	PDE3A	phosphodiesterase 3A, cGMP-inhibited	Multiple_Complex
TC1000007	8.45	10.33	-3.68	CREM	cAMP responsive element modulator	Multiple_Complex
TC0700008	5.75	7.63	-3.67	BUD31	Transcript Identified by AceView, Entrez Gene ID(s) 8896	Unassigned
TC0800008	7.64	9.42	-3.45	CA3	carbonic anhydrase III	Multiple_Complex
TC0800008	4.86	6.56	-3.26	MAL2	mal, T-cell differentiation protein 2 (gene/pseudogene)	Multiple_Complex
TC0400012	3.27	4.95	-3.2	FBXO8	Transcript Identified by AceView, Entrez Gene ID(s) 26269	Unassigned
TC1900007	4.37	6.04	-3.19	ZNF345	zinc finger protein 345	Multiple_Complex
TC1400007	4.47	6.13	-3.16	SAMD15	sterile alpha motif domain containing 15	Coding
TC1200008	3.37	5.01	-3.14	CCDC63	coiled-coil domain containing 63	Multiple_Complex
TC0X00006	2.82	4.39	-2.98	TBL1X	transducin (beta)-like 1X-linked	Multiple_Complex
TC0500010	3.04	4.62	-2.97	LIFR	leukemia inhibitory factor receptor alpha	Multiple_Complex
TC0200008	4.34	5.88	-2.91	PLGLB2	plasminogen-like B2	Multiple_Complex
TC1800006	9.85	11.39	-2.9	APCDD1	adenomatosis polyposis coli down-regulated 1	Multiple_Complex
TC1900010	4.32	5.85	-2.89	ZNF506	Transcript Identified by AceView, Entrez Gene ID(s) 284440; 4405	Unassigned
TC1400009	4.8	6.33	-2.88	GCH1	GTP cyclohydrolase 1	Multiple_Complex
TC1100010	3.33	4.85	-2.87	ASCL3	achaete-scute family bHLH transcription factor 3	Coding
TC0300013	4.55	6.07	-2.86	TFRC	Transcript Identified by AceView, Entrez Gene ID(s) 7037	Unassigned
TC0100016	6.42	7.94	-2.86	RGS5	regulator of G-protein signaling 5	Multiple_Complex
TC0300006	4.24	5.75	-2.84	CMTM8	CKLF-like MARVEL transmembrane domain containing 8	Coding
TC0X00011	4.01	5.51	-2.84	USP26	ubiquitin specific peptidase 26	Coding
TC1600011	8.08	9.58	-2.83	CDH3	cadherin 3, type 1, P-cadherin (placental)	Multiple_Complex
TC0300013	3.84	5.31	-2.76	TDGF1	teratocarcinoma-derived growth factor 1	Multiple_Complex
TC2200006	4.9	6.37	-2.76	GNAZ	guanine nucleotide binding protein (G protein), alpha z polypepti	Multiple_Complex
TC0X00007	3.25	4.72	-2.76	FAM133A	family with sequence similarity 133, member A	Coding
TC1100012	3.57	5.03	-2.75	IL18	interleukin 18	Multiple_Complex
TC1400009	3.25	4.7	-2.74	C14orf39	chromosome 14 open reading frame 39	Coding
TC0500008	6.82	8.27	-2.73	PCDHGC3; PCDHGA12; PCDHGB4; PC	protocadherin gamma subfamily C, 3; protocadherin gamma subf	Multiple_Complex
TC1800006	6.31	7.76	-2.73	TAF4B	TAF4b RNA polymerase II, TATA box binding protein (TBP)-associa	Multiple_Complex
TC0600008	4.57	6.01	-2.73	TJAP1	Zhang2013 ALT_ACCEPTOR, ALT_DONOR, coding, INTERNAL, intr	NonCoding
TC0400008	7.36	8.81	-2.73	PLK4	polo-like kinase 4	Multiple_Complex
TC0100009	5.55	7	-2.72	ABCD3	Transcript Identified by AceView, Entrez Gene ID(s) 5825	Unassigned
TC0200007	4.3	5.74	-2.69	EPCAM	epithelial cell adhesion molecule	Multiple_Complex
TC0200008	6.64	8.07	-2.69	TTC31	Memczak2013 ALT_ACCEPTOR, ALT_DONOR, coding, INTERNAL, i	NonCoding
TC0600007	3.83	5.25	-2.68	MUC22	mucin 22	Multiple_Complex
TC2200008	4.38	5.79	-2.66	RFPL2	ret finger protein-like 2	Multiple_Complex

TC0600007	4.28	5.69	-2.66	HCG9	HLA complex group 9 (non-protein coding)	Multiple_Complex
TC0100018	6.3	7.71	-2.66	ARHGAP29	Rho GTPase activating protein 29	Multiple_Complex
TC0300007	5.13	6.53	-2.65	UBA7	Memczak2013 ANTISENSE, coding, INTERNAL, intronic best transc	NonCoding
TSUnmapp	4.68	6.08	-2.65	OBP2B	odorant binding protein 2B	Coding
TC2200009	3.91	5.32	-2.64	SPECC1L-ADORA2A	SPECC1L-ADORA2A readthrough (NMD candidate)	Multiple_Complex
TC0200009	6.32	7.72	-2.64	CDCA7	cell division cycle associated 7	Multiple_Complex
TC1100010	3.52	4.92	-2.64	CAT	Memczak2013 ANTISENSE, CDS, coding, INTERNAL, UTR3 best tra	NonCoding
TC1100010	4.28	5.67	-2.63	CCDC34	Transcript Identified by AceView, Entrez Gene ID(s) 91057	Unassigned
TC1000007	3.95	5.34	-2.63	ANKRD30A	ankyrin repeat domain 30A	Multiple_Complex
TSUnmapp	4.08	5.47	-2.62	SERTAD4	SERTA domain containing 4	NonCoding
TC0900008	4.84	6.22	-2.61	C9orf43	chromosome 9 open reading frame 43	Multiple_Complex
TC1900007	9.86	11.24	-2.6	RAD23A	RAD23 homolog A, nucleotide excision repair protein	Multiple_Complex
TC0600011	5.81	7.19	-2.59	HIST1H1A	histone cluster 1, H1a	Coding
TC0900011	8.27	9.64	-2.59	FKBP15	FK506 binding protein 15	Multiple_Complex
TC0500008	5.5	6.87	-2.58	CAMK4	calcium/calmodulin-dependent protein kinase IV	Multiple_Complex
TC0X00010	2.23	3.59	-2.57	SOX3	SRY box 3	Multiple_Complex
TC0X00011	8.29	9.65	-2.57	FAM58A	family with sequence similarity 58, member A	Multiple_Complex
TC1500010	5.1	6.46	-2.56	CHRNB4	cholinergic receptor, nicotinic beta 4	Multiple_Complex
TC1300007	5.52	6.87	-2.55	KLF5	Kruppel-like factor 5 (intestinal)	Multiple_Complex
TC2000008	7.46	8.81	-2.55	MKKS	McKusick-Kaufman syndrome	Multiple_Complex
TC1900011	6.07	7.41	-2.55	RAB4B; MIA-RAB4B; RAB4B-EGLN2	RAB4B, member RAS oncogene family; MIA-RAB4B readthrough (	Multiple_Complex
TC0600011	6.35	7.7	-2.55	HIST1H4C	Jeck2013 ANTISENSE, CDS, coding, INTERNAL, OVCODE, OVEXON,	NonCoding
TC0X00010	3.83	5.18	-2.54	NXF5	nuclear RNA export factor 5	Multiple_Complex
TC0200008	4.15	5.48	-2.52	ZAP70	zeta chain of T cell receptor associated protein kinase 70kDa	Multiple_Complex
TC0800009	4.72	6.05	-2.51	DEFB136	defensin, beta 136	Coding
TC1100008	8.42	9.73	-2.48	DGAT2	diacylglycerol O-acyltransferase 2	Multiple_Complex
TC0X00008	5.02	6.33	-2.48	MTM1	myotubularin 1	Multiple_Complex
TC1400008	5.37	6.68	-2.48	SLC24A4	solute carrier family 24 (sodium/potassium/calcium exchanger),	Multiple_Complex
TC1700009	5.46	6.77	-2.47	SLC25A35	solute carrier family 25, member 35	Multiple_Complex
TC0400009	6.82	8.12	-2.46	MND1	meiotic nuclear divisions 1	Multiple_Complex
TC2000006	6.77	8.07	-2.46	SNAP25	synaptosome associated protein 25kDa	Multiple_Complex
TC0900009	11.62	12.91	-2.44	PAEP	progestagen-associated endometrial protein	Multiple_Complex
TC0700011	11.02	12.3	-2.43	SEMA3C	sema domain, immunoglobulin domain (Ig), short basic domain, s	Multiple_Complex
TC0700010	9.25	10.53	-2.43	CPVL	carboxypeptidase, vitellogenic-like	Multiple_Complex
TC0800006	7.58	8.85	-2.42	LPL	lipoprotein lipase	Multiple_Complex
TC0100018	3.83	5.1	-2.42	LEMD1	LEM domain containing 1	Multiple_Complex
TC1400009	4.88	6.14	-2.4	ADAM20	ADAM metallopeptidase domain 20	Coding
TC1100008	4.61	5.87	-2.4	OMP	olfactory marker protein	Coding
TC0600011	4.5	5.76	-2.4	BTBD9	Memczak2013 ALT_ACCEPTOR, ALT_DONOR, coding, INTERNAL, i	NonCoding
TC1400007	5.46	6.72	-2.39	SMOC1	SPARC related modular calcium binding 1	Multiple_Complex
TC1700010	6.65	7.91	-2.38	PSMC3IP	PSMC3 interacting protein	Multiple_Complex
TC0200012	5.79	7.04	-2.38	CCDC121	coiled-coil domain containing 121	Coding
TC0400010	9.71	10.96	-2.38	PPARGC1A	peroxisome proliferator-activated receptor gamma, coactivator 1	Multiple_Complex
TC1200010	6.66	7.91	-2.37	TIMELESS	timeless circadian clock	Multiple_Complex
TC0400012	5.67	6.92	-2.37	SLC39A8	solute carrier family 39 (zinc transporter), member 8	Multiple_Complex
TC0400009	3.39	4.63	-2.36	CCDC96	coiled-coil domain containing 96	Coding
TC1700010	7.79	9.03	-2.36	NR1D1	nuclear receptor subfamily 1, group D, member 1	Multiple_Complex

TC1100011	5.91	7.14	-2.35 UCP3	uncoupling protein 3 (mitochondrial, proton carrier)	Multiple_Complex
TC1900010	6.45	7.68	-2.35 CERS1; GDF1	ceramide synthase 1; growth differentiation factor 1	Multiple_Complex
TC1000009	8.06	9.29	-2.35 NUDT5	nudix hydrolase 5	Multiple_Complex
TC1700006	6.06	7.29	-2.34 RAP1GAP2	RAP1 GTPase activating protein 2	Multiple_Complex
TC1300007	3.44	4.66	-2.34 OLFM4	olfactomedin 4	Coding
TC0100008	4.14	5.36	-2.33 TMEM125	transmembrane protein 125	Multiple_Complex
TC0100014	6.33	7.55	-2.32 SETSIP	SET-like protein	Coding
TC1500010	7.04	8.25	-2.32 IVD	isovaleryl-CoA dehydrogenase	Multiple_Complex
TC0700009	5.11	6.32	-2.32 EN2	engrailed homeobox 2	Coding
TC1000008	3.57	4.78	-2.32 ABCC2	ATP binding cassette subfamily C member 2	Multiple_Complex
TC1000009	4.56	5.77	-2.32 CCDC3	coiled-coil domain containing 3	Multiple_Complex
TC1600006	3.25	4.45	-2.31 A2BP1	Transcript Identified by AceView, Entrez Gene ID(s) 54715	Unassigned
TC0500010	7.31	8.51	-2.31 RAD1	RAD1 checkpoint DNA exonuclease	Multiple_Complex
TC0200011	7.45	8.65	-2.3 E2F6	E2F transcription factor 6	Multiple_Complex
TC1100008	4.27	5.47	-2.29 LINC01537	long intergenic non-protein coding RNA 1537	NonCoding
TC0100011	3.57	4.76	-2.29 IL24	interleukin 24	Multiple_Complex
TC1100013	3.42	4.61	-2.29 ZBED5	zinc finger, BED-type containing 5	NonCoding
TC1700010	6.03	7.22	-2.28 B9D1	B9 protein domain 1	Multiple_Complex
TC0800009	3.32	4.51	-2.27 PRR23D2	proline rich 23 domain containing 2	Coding
TC0800011	8.19	9.37	-2.27 ATAD2	ATPase family, AAA domain containing 2	Multiple_Complex
TC2000006	6.5	7.67	-2.26 ISM1	isthmin 1, angiogenesis inhibitor	Coding
TC1100007	4.84	6.02	-2.26 TCP11L1	t-complex 11, testis-specific-like 1	Multiple_Complex
TC1100012	3.82	4.99	-2.25 SORL1	Memczak2013 ANTISENSE, CDS, coding, INTERNAL best transcript	NonCoding
TC1700012	4.07	5.23	-2.25 SPAG5	sperm associated antigen 5	Multiple_Complex
TC0700013	3.65	4.81	-2.24 NME8	NME/NM23 family member 8	Multiple_Complex
TC1400010	3.17	4.33	-2.23 ASPG	asparaginase	Multiple_Complex
TC0500007	3.63	4.78	-2.23 RAB3C	RAB3C, member RAS oncogene family	Multiple_Complex
TC0700007	6.88	8.03	-2.22 WIPF3	WAS/WASL interacting protein family, member 3	Multiple_Complex
TC0100006	6.12	7.27	-2.22 CPTP	ceramide-1-phosphate transfer protein	Multiple_Complex
TC1400006	3.83	4.98	-2.22 SLC39A2	solute carrier family 39 (zinc transporter), member 2	Multiple_Complex
TC0800008	7.22	8.38	-2.22 SDC2	syndecan 2	Multiple_Complex
TC1300006	3.68	4.83	-2.22 RGCC	regulator of cell cycle	Multiple_Complex
TC0X00008	3.49	4.64	-2.21 VGLL1	vestigial-like family member 1	Multiple_Complex
TC0200012	4.39	5.54	-2.21 ZNF512	Memczak2013 ANTISENSE, coding, INTERNAL, UTR3 best transcri	NonCoding
TSUnmapp	4.79	5.93	-2.21 HMBS	hydroxymethylbilane synthase	NonCoding
TC0600013	8.89	10.03	-2.2 SOD2	superoxide dismutase 2, mitochondrial	Multiple_Complex
TC0600011	3.53	4.66	-2.2 PXT1	peroxisomal, testis specific 1	Coding
TC0X00010	4.14	5.28	-2.2 RPS4X	Zhang2013 ALT_ACCEPTOR, ALT_DONOR, coding, INTERNAL, intr	NonCoding
TC1800008	5.42	6.55	-2.2 PTPN2	Transcript Identified by AceView, Entrez Gene ID(s) 5771	Coding
TC0400009	9.28	10.41	-2.2 STX18	syntaxin 18	Multiple_Complex
TC0300009	9.43	10.56	-2.2 RSRC1	arginine/serine-rich coiled-coil 1	Multiple_Complex
TC0900012	7.56	8.69	-2.19 PLIN2	perilipin 2	Multiple_Complex
TC1300009	4.35	5.49	-2.19 SLC15A1	solute carrier family 15 (oligopeptide transporter), member 1	Multiple_Complex
TC0800011	6.18	7.31	-2.19 CCNE2	cyclin E2	Multiple_Complex
TC0700012	9.81	10.94	-2.18 VGF	VGF nerve growth factor inducible	Coding
TC0700012	5.68	6.81	-2.18 NRCAM	neuronal cell adhesion molecule	Multiple_Complex
TC0X00011	5.82	6.94	-2.18 SLC10A3	solute carrier family 10, member 3	Coding

TC1500009	4.08	5.2	-2.18	UBAP1L	ubiquitin associated protein 1 like	Multiple_Complex
TC1600007	6.92	8.05	-2.18	NKD1	naked cuticle homolog 1 (Drosophila)	Multiple_Complex
TC0100008	5.51	6.64	-2.18	CYR61	cysteine-rich, angiogenic inducer, 61	Multiple_Complex
TC1700011	4.83	5.95	-2.18	FAM117A	family with sequence similarity 117, member A	Multiple_Complex
TC1100010	5.4	6.52	-2.18	ACP2	acid phosphatase 2, lysosomal	Multiple_Complex
TC0100008	7.15	8.27	-2.17	CDC20	cell division cycle 20	Multiple_Complex
TC1500010	5.26	6.38	-2.17	TLN2	talin 2	NonCoding
TC1000012	9.6	10.72	-2.17	AS3MT	arsenite methyltransferase	Multiple_Complex
TC0200008	9.05	10.16	-2.16	KCNIP3	Kv channel interacting protein 3, calsenilin	Multiple_Complex
TC0600008	5.26	6.37	-2.16	KLC4	kinesin light chain 4	Multiple_Complex
TC1000008	6.35	7.46	-2.16	CEP55	centrosomal protein 55kDa	Multiple_Complex
TC0300010	6.48	7.58	-2.16	ELP6	elongator acetyltransferase complex subunit 6	Multiple_Complex
TC1300007	9.08	10.18	-2.16	CKAP2	cytoskeleton associated protein 2	Multiple_Complex
TC0300012	7.47	8.57	-2.15	CCDC191	coiled-coil domain containing 191	Multiple_Complex
TC1900009	3.83	4.94	-2.15	LINGO3	leucine rich repeat and Ig domain containing 3	Coding
TC0300006	5.29	6.39	-2.15	EDEM1	Transcript Identified by AceView, Entrez Gene ID(s) 9695	Unassigned
TC0100007	6	7.1	-2.15	GPR3	G protein-coupled receptor 3	Coding
TC0500009	7.4	8.5	-2.14	TCERG1	Transcript Identified by AceView, Entrez Gene ID(s) 10915	Unassigned
TC1100012	4.5	5.59	-2.14	BLID; MIR100HG	BH3-like motif containing, cell death inducer; mir-100-let-7a-2 clu	Multiple_Complex
TC0800010	11.97	13.06	-2.13	HEY1	hes-related family bHLH transcription factor with YRPW motif 1	Multiple_Complex
TC1800008	7.96	9.05	-2.13	RTTN	rotatin	Multiple_Complex
TC0300007	3.4	4.49	-2.13	ARPP21	Transcript Identified by AceView, Entrez Gene ID(s) 10777	Unassigned
TC0100012	4.04	5.13	-2.13	OR2G3	olfactory receptor, family 2, subfamily G, member 3	Coding
TC1300008	4.33	5.42	-2.13	CPB2	carboxypeptidase B2 (plasma)	Coding
TC0600010	7.09	8.17	-2.12	ACAT2	acetyl-CoA acetyltransferase 2	Multiple_Complex
TC0800008	8.77	9.85	-2.12	SQLE	squalene epoxidase	Multiple_Complex
TC2000008	4.12	5.2	-2.12	CST9	cystatin 9 (testatin)	Multiple_Complex
TC1500010	3.61	4.69	-2.12	RLBP1	retinaldehyde binding protein 1	Multiple_Complex
TC1200008	4.05	5.13	-2.12	LOC101928137; RP11-314D7.2	uncharacterized LOC101928137; novel transcript	NonCoding
TC1700007	9.18	10.26	-2.12	CDC6	cell division cycle 6	Multiple_Complex
TC1400008	8.49	9.57	-2.11	RNASE1	ribonuclease, RNase A family, 1 (pancreatic)	Multiple_Complex
TC2200006	7.25	8.33	-2.11	ADRBK2	adrenergic, beta, receptor kinase 2	Multiple_Complex
TSUnmapp	4.57	5.65	-2.11	INPP5D	inositol polyphosphate-5-phosphatase D	NonCoding
TC1200012	5.2	6.28	-2.11	ACSS3	acyl-CoA synthetase short-chain family member 3	Multiple_Complex
TC1700009	3.73	4.81	-2.1	ALOXE3	arachidonate lipoxygenase 3	Multiple_Complex
TC0600010	4.88	5.95	-2.1	GFOD1	glucose-fructose oxidoreductase domain containing 1	Multiple_Complex
TC0900011	4.1	5.17	-2.1	C9orf116	chromosome 9 open reading frame 116	Multiple_Complex
TC0300013	7.12	8.19	-2.1	LRRC34	leucine rich repeat containing 34	Multiple_Complex
TC0200012	3.65	4.72	-2.1	SULT6B1	sulfotransferase family 6B member 1	Multiple_Complex
TC0200014	11.19	12.26	-2.1	CXCR4	chemokine (C-X-C motif) receptor 4	Multiple_Complex
TC0200009	6.04	7.11	-2.1	STEAP3	STEAP family member 3, metalloreductase	Multiple_Complex
TC0200016	9.74	10.81	-2.1	LRRFIP1	leucine rich repeat (in FLII) interacting protein 1	Multiple_Complex
TC0600012	9.7	10.77	-2.09	NDUFAF4	NADH dehydrogenase (ubiquinone) complex I, assembly factor 4	Multiple_Complex
TC1000011	4.26	5.31	-2.08	KLLN	killin, p53-regulated DNA replication inhibitor	Coding
TC0400008	7.7	8.76	-2.08	MRPS18C	mitochondrial ribosomal protein S18C	Multiple_Complex
TC0700013	4.45	5.5	-2.08	HOXA6	homeobox A6	Multiple_Complex
TC1400009	6.95	8.01	-2.08	POLE2	polymerase (DNA directed), epsilon 2, accessory subunit	Multiple_Complex

TC1000010	5.93	6.98	-2.08	ASCC1	Transcript Identified by AceView, Entrez Gene ID(s) 51008	Coding
TC0400010	4.09	5.14	-2.07	LRRC66	leucine rich repeat containing 66	Coding
TC1000006	9.1	10.15	-2.07	COMMD3-BMI1; BMI1; COMMD3	COMMD3-BMI1 readthrough; BMI1 proto-oncogene, polycomb ri	Multiple_Complex
TC1700010	2.7	3.75	-2.07	PRR15L	proline rich 15-like	Coding
TC2200006	8.24	9.29	-2.07	CDC45	cell division cycle 45	Multiple_Complex
TC1500008	6.9	7.95	-2.07	ISG20	interferon stimulated exonuclease gene 20kDa	Multiple_Complex
TC1300008	5.08	6.13	-2.07	ESD	Transcript Identified by AceView, Entrez Gene ID(s) 2098	Unassigned
TC2000009	6.4	7.45	-2.07	SULF2	sulfatase 2	Multiple_Complex
TC2200006	7.17	8.22	-2.07	GP1BB; SEPT5	glycoprotein Ib (platelet), beta polypeptide; septin 5	Multiple_Complex
TC0800012	5.61	6.65	-2.07	SORBS3	sorbin and SH3 domain containing 3	Multiple_Complex
TC0700008	9.58	10.63	-2.07	DBF4	DBF4 zinc finger	Multiple_Complex
TC2100007	3.19	4.24	-2.07	FAM3B	family with sequence similarity 3, member B	Multiple_Complex
TC0600014	3.02	4.07	-2.06	OR5V1	olfactory receptor, family 5, subfamily V, member 1	Coding
TC1900011	3.24	4.28	-2.06	SULT2A1	sulfotransferase family 2A member 1	Coding
TC0400012	4.47	5.51	-2.06	FRG2	FSHD region gene 2	Coding
TC0200008	5.12	6.16	-2.06	ACOXL	acyl-CoA oxidase-like	Multiple_Complex
TC1100006	4.03	5.08	-2.06	OR52E1	olfactory receptor, family 52, subfamily E, member 1 (gene/pseud	Pseudogene
TC0X00009	3.44	4.48	-2.06	XAGE1E; XAGE1B	X antigen family, member 1E; X antigen family, member 1B	Multiple_Complex
TC0400008	9.58	10.62	-2.06	HSPA4L	heat shock 70kDa protein 4-like	Multiple_Complex
TC0100015	3.32	4.36	-2.06	FLG2	filaggrin family member 2	Coding
TC0100014	4.31	5.35	-2.06	F3	coagulation factor III (thromboplastin, tissue factor)	Multiple_Complex
TC2100006	5.01	6.05	-2.05	CLIC6	chloride intracellular channel 6	Coding
TC1700010	5.87	6.9	-2.05	LGALS9B	lectin, galactoside-binding, soluble, 9B	Multiple_Complex
TC0400006	5.13	6.16	-2.05	LAP3	Transcript Identified by AceView, Entrez Gene ID(s) 51056	Unassigned
TC1000008	8.24	9.27	-2.05	PPIF	peptidylprolyl isomerase F	Multiple_Complex
TC1900006	6.32	7.35	-2.05	ATP5D	ATP synthase, H+ transporting, mitochondrial F1 complex, delta s	Multiple_Complex
TC1600009	9.69	10.72	-2.04	HAGH	hydroxyacylglutathione hydrolase	Multiple_Complex
TC0300007	5.69	6.72	-2.04	LARS2	leucyl-tRNA synthetase 2, mitochondrial	Multiple_Complex
TC0100013	6.85	7.88	-2.04	RPA2	replication protein A2	Multiple_Complex
TC0100015	6	7.03	-2.04	IL6R	Memczak2013 ANTISENSE, coding, INTERNAL, UTR3 best transcri	NonCoding
TC1400006	7.7	8.73	-2.04	PARP2	poly(ADP-ribose) polymerase 2	Multiple_Complex
TC1600009	7.01	8.04	-2.04	SEPHS2	selenophosphate synthetase 2	Coding
TC1900007	6.86	7.89	-2.04	NUDT19	nudix hydrolase 19	Multiple_Complex
TC0300013	5.5	6.52	-2.04	SUMF1	sulfatase modifying factor 1	NonCoding
TC1600009	4.3	5.32	-2.03	CARHSP1	calcium regulated heat stable protein 1	Multiple_Complex
TC0400008	5.32	6.35	-2.03	LOC105377348; RP11-10L12.4; UBE2	uncharacterized LOC105377348; Salzman2013 ANTISENSE, codin	NonCoding
TC0200014	5.27	6.29	-2.03	NR4A2	nuclear receptor subfamily 4, group A, member 2	Multiple_Complex
TC1200006	4.48	5.5	-2.03	KLRF1	killer cell lectin-like receptor subfamily F, member 1	Multiple_Complex
TC0X00007	4.38	5.4	-2.03	MAGED4B; MAGED4; SNORA11D	melanoma antigen family D4B; melanoma antigen family D4; sma	Multiple_Complex
TC1600008	5.85	6.87	-2.02	NUDT7	nudix hydrolase 7	Multiple_Complex
TC0600012	4.67	5.68	-2.02	SNAP91	synaptosome associated protein 91kDa	Multiple_Complex
TC0700010	3.64	4.65	-2.02	AGR2	anterior gradient 2, protein disulphide isomerase family member	Multiple_Complex
TC1900011	7.2	8.21	-2.02	GRWD1	glutamate-rich WD repeat containing 1	Multiple_Complex
TC1000012	8.15	9.16	-2.02	ENTPD1	ectonucleoside triphosphate diphosphohydrolase 1	Multiple_Complex
TC1700012	5.59	6.6	-2.02	AXIN2	axin 2	Multiple_Complex
TC1300009	3.13	4.13	-2.01	FAM155A	Jeck2013 ALT_ACCEPTOR, ALT_DONOR, coding, INTERNAL, intron	NonCoding
TC0800008	5.55	6.55	-2.01	SPAG1	sperm associated antigen 1	Multiple_Complex

TC0X00010	3.38	4.39	-2.01	SERPINA7	serpin peptidase inhibitor, clade A (alpha-1 antiproteinase, antitr	Multiple_Complex
TC0500010	6.12	7.13	-2.01	C5orf34	chromosome 5 open reading frame 34	Multiple_Complex
TC1900008	7.04	8.05	-2	ZNF175	zinc finger protein 175	Multiple_Complex
TC1700009	5.34	6.34	-2	RASD1	RAS, dexamethasone-induced 1	Coding
TC0100015	5.11	6.11	-2	DPYD	dihydropyrimidine dehydrogenase	Multiple_Complex
TC0100013	4.89	5.89	-2	ZBTB17	zinc finger and BTB domain containing 17	Multiple_Complex
TC0300011	4.86	5.86	-1.99	BBX	Memczak2013 ANTISENSE, coding, INTERNAL, intronic best transc	NonCoding
TC0700013	3.55	4.54	-1.99	HTR5A-AS1	HTR5A antisense RNA 1	NonCoding
TC1100010	6.18	7.17	-1.99	ARFGAP2	ADP-ribosylation factor GTPase activating protein 2	Multiple_Complex
TC0700006	4.41	5.4	-1.99	IL6	interleukin 6	Multiple_Complex
TC0800011	7.18	8.18	-1.99	NCALD	neurocalcin delta	Multiple_Complex
TC0200016	6.92	7.91	-1.99	KLHL23	kelch-like family member 23	Multiple_Complex
TC1100007	4.31	5.3	-1.99	MS4A15	membrane-spanning 4-domains, subfamily A, member 15	Multiple_Complex
TC0900010	3.07	4.07	-1.99	SYK	Memczak2013 ANTISENSE, CDS, coding, INTERNAL best transcript	NonCoding
TC1400010	5.53	6.51	-1.98	ANKRD9	ankyrin repeat domain 9	Coding
TC0100010	8.38	9.37	-1.98	RGL1	ral guanine nucleotide dissociation stimulator-like 1	Multiple_Complex
TC0700012	10.09	11.07	-1.98	KIAA1549	KIAA1549	Multiple_Complex
TC0700011	8.75	9.74	-1.98	BCL7B	B-cell CLL/lymphoma 7B	Multiple_Complex
TC0300014	8.89	9.88	-1.98	COX17	COX17 cytochrome c oxidase copper chaperone	Multiple_Complex
TC1700009	7	7.98	-1.98	SAT2	spermidine/spermine N1-acetyltransferase family member 2	Multiple_Complex
TC2200008	4.95	5.93	-1.98	YDJC	YdjC homolog (bacterial)	Multiple_Complex
TC1200009	10.04	11.03	-1.98	FOXM1	forkhead box M1	Multiple_Complex
TC0300010	7.69	8.67	-1.97	SH3BP5	SH3-domain binding protein 5 (BTK-associated)	Multiple_Complex
TC1900010	8.24	9.22	-1.97	EXOSC5	exosome component 5	Multiple_Complex
TC2000008	8.34	9.32	-1.97	PPDPF	pancreatic progenitor cell differentiation and proliferation factor	Multiple_Complex
TC0100012	4.34	5.31	-1.97	KCNK1	potassium channel, two pore domain subfamily K, member 1	Multiple_Complex
TC0200010	6.09	7.06	-1.97	IGFBP2	insulin like growth factor binding protein 2	Multiple_Complex
TC1600010	10.6	11.58	-1.96	RANBP10	RAN binding protein 10	Multiple_Complex
TC0300008	6.09	7.06	-1.96	ALCAM	activated leukocyte cell adhesion molecule	Multiple_Complex
TC1900011	7.38	8.36	-1.96	NDUFA13	NADH dehydrogenase (ubiquinone) 1 alpha subcomplex, 13	Multiple_Complex
TC2200006	6.92	7.89	-1.96	RAB36	RAB36, member RAS oncogene family	Multiple_Complex
TC1100013	8.83	9.8	-1.96	CHEK1	checkpoint kinase 1	Multiple_Complex
TC1600007	3.18	4.15	-1.96	C16orf82	chromosome 16 open reading frame 82	Multiple_Complex
TSUnmapp	5.44	6.41	-1.96	CCDC84	coiled-coil domain containing 84	NonCoding
TC0200014	8.23	9.2	-1.96	LY75-CD302; CD302; LY75	LY75-CD302 readthrough; CD302 molecule; lymphocyte antigen 7	Multiple_Complex
TC1600011	6.2	7.17	-1.96	TMEM231	transmembrane protein 231	Multiple_Complex
TC0500013	6.97	7.94	-1.96	LCP2	lymphocyte cytosolic protein 2	Multiple_Complex
TC1000011	4.22	5.19	-1.96	HTR7	5-hydroxytryptamine (serotonin) receptor 7, adenylate cyclase-co	Multiple_Complex
TC1100012	4.46	5.42	-1.95	MMP7	matrix metallopeptidase 7	Multiple_Complex
TC0700008	8.31	9.27	-1.95	CLIP2	CAP-GLY domain containing linker protein 2	Multiple_Complex
TC0200016	7.01	7.97	-1.95	KLHL23	kelch-like family member 23	Multiple_Complex
TC1400009	6.55	7.51	-1.95	MAP3K9	mitogen-activated protein kinase kinase kinase 9	Multiple_Complex
TC2000008	6.35	7.31	-1.95	SLCO4A1	solute carrier organic anion transporter family, member 4A1	Multiple_Complex
TC0300009	3.7	4.66	-1.95	IL12A	interleukin 12A	Multiple_Complex
TC1400009	9.49	10.44	-1.94	TTC7B	tetratricopeptide repeat domain 7B	Multiple_Complex
TSUnmapp	4.36	5.32	-1.94	SLC2A6	solute carrier family 2 (facilitated glucose transporter), member 6	Coding
TC0700007	4.06	5.02	-1.94	WBSCR17	Williams-Beuren syndrome chromosome region 17	Multiple_Complex

TC1100006	4.89	5.84	-1.93	BRSK2	BR serine/threonine kinase 2	Multiple_Complex
TC1000012	8.95	9.9	-1.93	BORCS7-ASMT	BORCS7-ASMT readthrough (NMD candidate)	Multiple_Complex
TC0200012	7.37	8.32	-1.93	ZNF513	zinc finger protein 513	Multiple_Complex
TC0400010	3.32	4.27	-1.93	GABRA2	gamma-aminobutyric acid (GABA) A receptor, alpha 2	Multiple_Complex
TC0Y00006	4.8	5.74	-1.93	RPS4Y2	ribosomal protein S4, Y-linked 2	Coding
TC1200012	4.57	5.52	-1.93	ZNF891	zinc finger protein 891	Multiple_Complex
TC1900007	6.59	7.54	-1.93	ZNF714; VN1R81P	zinc finger protein 714; vomeronasal 1 receptor 81 pseudogene	Multiple_Complex
TC0900009	3.02	3.97	-1.93	IFNA7	interferon, alpha 7	Coding
TC1900011	4.66	5.6	-1.93	ZNF98	zinc finger protein 98	NonCoding
TC1500007	6.18	7.13	-1.93	PAQR5	progestin and adipoQ receptor family member V	Multiple_Complex
TC0200009	3.75	4.7	-1.93	MYO7B	myosin VIIB	Multiple_Complex
TC2000006	4.64	5.59	-1.92	SOX12	SRY box 12	Coding
TC0600006	9.01	9.95	-1.92	WRNIP1	Werner helicase interacting protein 1	Multiple_Complex
TC1300006	8.4	9.34	-1.92	TNFRSF19	tumor necrosis factor receptor superfamily, member 19	Multiple_Complex
TC0200016	3.88	4.82	-1.92	TMEM178A	transmembrane protein 178A	Multiple_Complex
TC1500007	8.21	9.15	-1.92	LOXL1	lysyl oxidase-like 1	Multiple_Complex
TC2200009	6.15	7.09	-1.92	CHKB-CPT1B	CHKB-CPT1B readthrough (NMD candidate)	Multiple_Complex
TC0400007	3.22	4.16	-1.92	PARM1	prostate androgen-regulated mucin-like protein 1	Multiple_Complex
TC1900011	6.44	7.38	-1.92	PPP1R37	protein phosphatase 1, regulatory subunit 37	Multiple_Complex
TSUnmapp	5.93	6.86	-1.92	VPS11	VPS11, CORVET/HOPS core subunit [Source:HGNC Symbol;Acc:HG	NonCoding
TC1100010	5.63	6.57	-1.92	LMO2	LIM domain only 2 (rhombotin-like 1)	Multiple_Complex
TC1100006	6.36	7.3	-1.92	SMPD1	sphingomyelin phosphodiesterase 1, acid lysosomal	Multiple_Complex
TC1900010	3.12	4.05	-1.91	CEACAM7	carcinoembryonic antigen-related cell adhesion molecule 7	Multiple_Complex
TC0400007	3.09	4.03	-1.91	SLC10A4	solute carrier family 10, member 4	Coding
TC0600008	8.63	9.57	-1.91	TTK	TTK protein kinase	Multiple_Complex
TC0300012	3.57	4.51	-1.91	ERICH6	glutamate rich 6	Multiple_Complex
TC1200006	5.19	6.12	-1.91	PRH2	proline-rich protein HaeIII subfamily 2	Coding
TC0900010	10.6	11.53	-1.91	GOLM1	golgi membrane protein 1	Multiple_Complex
TC0400006	7.36	8.29	-1.91	TACC3	transforming, acidic coiled-coil containing protein 3	Multiple_Complex
TSUnmapp	4.92	5.85	-1.91	OBP2B	odorant binding protein 2B	NonCoding
TC1700009	3.8	4.73	-1.91	HS3ST3A1	Jeck2013 ALT_ACCEPTOR, ALT_DONOR, coding, INTERNAL, intron	NonCoding
TC2200008	7.84	8.77	-1.9	RANGAP1	Ran GTPase activating protein 1	Multiple_Complex
TC0300008	4.39	5.32	-1.9	CEP97	Transcript Identified by AceView, Entrez Gene ID(s) 79598	Unassigned
TC0200007	4.93	5.86	-1.9	VAX2	ventral anterior homeobox 2	Coding
TC0800011	3.95	4.88	-1.9	SNX31	sorting nexin 31	Multiple_Complex
TC0100010	7.04	7.97	-1.9	PPOX	protoporphyrinogen oxidase	Multiple_Complex
TC0100014	7.63	8.55	-1.9	DNTTIP2	deoxynucleotidyltransferase, terminal, interacting protein 2	Multiple_Complex
TC1500008	3.76	4.69	-1.9	GABRB3	gamma-aminobutyric acid (GABA) A receptor, beta 3	Multiple_Complex
TC1100011	4.89	5.82	-1.9	PRPF19	Memczak2013 ALT_ACCEPTOR, ALT_DONOR, coding, INTERNAL, i	NonCoding
TC1900012	8.02	8.95	-1.9	ETFB	electron-transfer-flavoprotein, beta polypeptide	Multiple_Complex
TC1900009	8.42	9.34	-1.9	SGTA	small glutamine-rich tetratricopeptide repeat (TPR)-containing, al	Multiple_Complex
TC0X00007	9.38	10.3	-1.9	MAGEH1	MAGE family member H1	Coding
TC1100009	4.05	4.97	-1.89	OR52H1	olfactory receptor, family 52, subfamily H, member 1	Coding
TC2100007	5.04	5.96	-1.89	KRTAP6-1	keratin associated protein 6-1	Coding
TC0100014	8.03	8.95	-1.89	DEPDC1	DEP domain containing 1	Multiple_Complex
TC1100008	5.56	6.48	-1.89	KLC2	kinesin light chain 2	Multiple_Complex
TC0100017	5.28	6.2	-1.89	PIK3C2B	phosphatidylinositol-4-phosphate 3-kinase, catalytic subunit type	Multiple_Complex

TC0800011	7.74	8.66	-1.89	HAS2	hyaluronan synthase 2	Multiple_Complex
TC0200007	6.73	7.65	-1.89	CHAC2	ChaC, cation transport regulator homolog 2 (E. coli)	Coding
TC0600011	9.99	10.91	-1.89	YIPF3	Yip1 domain family member 3	Multiple_Complex
TC1000010	8.04	8.95	-1.89	PPA1	pyrophosphatase (inorganic) 1	Multiple_Complex
TC0700011	9.04	9.96	-1.89	ZNF680	zinc finger protein 680	Multiple_Complex
TC0500009	5.54	6.46	-1.89	ZDHHC11	Transcript Identified by AceView, Entrez Gene ID(s) 79844	Unassigned
TC1300008	9.73	10.65	-1.89	MED4	mediator complex subunit 4	Multiple_Complex
TC2000009	3.41	4.33	-1.89	CNBD2	cyclic nucleotide binding domain containing 2	Coding
TC1300008	9.1	10.01	-1.89	ATP11A	ATPase, class VI, type 11A	Multiple_Complex
TC0600007	6.92	7.83	-1.88	PIM1	Pim-1 proto-oncogene, serine/threonine kinase	Multiple_Complex
TC1100007	8.04	8.95	-1.88	SLC39A13	solute carrier family 39 (zinc transporter), member 13	Multiple_Complex
TC0X00007	6.18	7.09	-1.88	KIF4A	kinesin family member 4A	Multiple_Complex
TC0500012	3	3.92	-1.88	HAVCR1	hepatitis A virus cellular receptor 1	Multiple_Complex
TC0800009	3.66	4.57	-1.88	FGL1	fibrinogen like 1	Multiple_Complex
TC0300013	7.63	8.54	-1.88	ROBO2	roundabout guidance receptor 2	Multiple_Complex
TC0800012	3.9	4.81	-1.88	CCDC166	coiled-coil domain containing 166	Coding
TC1400009	7.16	8.07	-1.88	FBLN5	fibulin 5	Multiple_Complex
TC1500009	5.96	6.87	-1.88	CA12	carbonic anhydrase XII	Multiple_Complex
TC2000007	8.56	9.46	-1.88	FAM83D	family with sequence similarity 83, member D	Multiple_Complex
TC0200007	3.78	4.69	-1.88	CCDC85A	Transcript Identified by AceView, Entrez Gene ID(s) 114800	Unassigned
TC1400008	6.93	7.83	-1.88	OSGEP	O-sialoglycoprotein endopeptidase	Multiple_Complex
TC1700007	4.06	4.96	-1.88	AOC3	amine oxidase, copper containing 3	Multiple_Complex
TC1900008	3.55	4.45	-1.87	CBLC	Cbl proto-oncogene C, E3 ubiquitin protein ligase	Multiple_Complex
TC0100013	5.43	6.34	-1.87	TMEM53	transmembrane protein 53	Multiple_Complex
TC1900006	6.61	7.51	-1.87	C19orf66	chromosome 19 open reading frame 66	Multiple_Complex
TC0400011	10.09	10.99	-1.87	CCNA2	cyclin A2	Multiple_Complex
TC0500008	5.96	6.86	-1.87	SLF1	SMC5-SMC6 complex localization factor 1	Multiple_Complex
TC0100017	7.22	8.13	-1.87	RAB29	RAB29, member RAS oncogene family	Multiple_Complex
TC2000008	9.62	10.52	-1.87	TPD52L2	tumor protein D52-like 2	Multiple_Complex
TC1300008	7.92	8.82	-1.87	SKA3	spindle and kinetochore associated complex subunit 3	Multiple_Complex
TC1100010	6.42	7.32	-1.87	E2F8	E2F transcription factor 8	Multiple_Complex
TSUnmapp	3.69	4.59	-1.87	SAG	S-antigen; retina and pineal gland (arrestin)	NonCoding
TC2000006	7.5	8.4	-1.87	TRIB3	tribbles pseudokinase 3	Multiple_Complex
TC0600007	4.85	5.75	-1.87	OR2H1	olfactory receptor, family 2, subfamily H, member 1	Multiple_Complex
TC1400008	8.9	9.8	-1.87	RABGGTA	Rab geranylgeranyltransferase, alpha subunit	Multiple_Complex
TC1600008	11.66	12.56	-1.87	HSBP1	heat shock factor binding protein 1	Multiple_Complex
TC0400006	4.38	5.27	-1.86	USP17L18; USP17L11	ubiquitin specific peptidase 17-like family member 18; ubiquitin s	Coding
TC0600007	12.07	12.97	-1.86	SRSF3	serine/arginine-rich splicing factor 3	Multiple_Complex
TSUnmapp	3.31	4.21	-1.86	HMBS	hydroxymethylbilane synthase	NonCoding
TC1000008	11.49	12.39	-1.86	SMC3	structural maintenance of chromosomes 3	Multiple_Complex
TC0700010	6.63	7.52	-1.86	MYL7	myosin light chain 7	Multiple_Complex
TC2100007	4.99	5.89	-1.86	NDUFV3	Transcript Identified by AceView, Entrez Gene ID(s) 4731	Unassigned
TC1700012	8.18	9.07	-1.86	HN1	hematological and neurological expressed 1	Multiple_Complex
TSUnmapp	4.68	5.57	-1.86	SERTAD4	SERTA domain containing 4	Coding
TC0X00006	9.03	9.92	-1.86	SCML1	sex comb on midleg-like 1 (Drosophila)	Multiple_Complex
TC1500010	7.64	8.53	-1.86	HOMER2	homer scaffolding protein 2	Multiple_Complex
TC0300011	6.39	7.28	-1.86	SLC25A20	solute carrier family 25 (carnitine/acylcarnitine translocase), mem	Multiple_Complex

TC0600013	7.07	7.97	-1.86 FBXO5	F-box protein 5	Multiple_Complex
TC0100011	5.08	5.97	-1.86 GPR37L1	G protein-coupled receptor 37 like 1	Multiple_Complex
TC0100010	4.55	5.45	-1.86 FASLG	Fas ligand (TNF superfamily, member 6)	Coding
TC2000009	5.34	6.24	-1.86 SYCP2	synaptonemal complex protein 2	Multiple_Complex
TC0X00007	5.17	6.06	-1.86 PABPC5	poly(A) binding protein, cytoplasmic 5	Coding
TC2100007	6.45	7.34	-1.85 DNAJC28	DnaJ (Hsp40) homolog, subfamily C, member 28	Coding
TC1000007	4.44	5.33	-1.85 ALOX5	Transcript Identified by AceView, Entrez Gene ID(s) 240	Unassigned
TC0400008	7.29	8.18	-1.85 TIGD2	tigger transposable element derived 2	Multiple_Complex
TSUnmapp	6.21	7.1	-1.85 TRAPPC4	trafficking protein particle complex 4	Coding
TC1800006	10.58	11.47	-1.85 GNAL	guanine nucleotide binding protein (G protein), alpha activating a	Multiple_Complex
TC1900010	6.33	7.21	-1.85 JUND	jun D proto-oncogene	Coding
TC2000009	5.57	6.46	-1.85 EFCAB8	EF-hand calcium binding domain 8	Coding
TC1900006	4.62	5.5	-1.85 NDUFS7	NADH dehydrogenase (ubiquinone) Fe-S protein 7, 20kDa (NADH	Multiple_Complex
TC0100007	9.38	10.27	-1.85 AKIRIN1	akirin 1	Multiple_Complex
TC0800010	6.89	7.77	-1.85 C8orf59	chromosome 8 open reading frame 59	Multiple_Complex
TC1200007	3.02	3.91	-1.85 ANKRD33	ankyrin repeat domain 33	Multiple_Complex
TC1100008	4.05	4.94	-1.85 TRIM77	tripartite motif containing 77	Coding
TC0800009	3.33	4.22	-1.85 CSMD1	Transcript Identified by AceView, Entrez Gene ID(s) 64478	Unassigned
TC0600008	6.6	7.49	-1.85 MYO6	Transcript Identified by AceView, Entrez Gene ID(s) 4646	Unassigned
TC0700013	7.65	8.54	-1.85 GCC1	GRIP and coiled-coil domain containing 1	Multiple_Complex
TC0900011	10.3	11.18	-1.85 MAPKAP1	mitogen-activated protein kinase associated protein 1	Multiple_Complex
TC0100015	5.98	6.86	-1.85 POGZ	Transcript Identified by AceView, Entrez Gene ID(s) 23126	Unassigned
TC0X00009	10	10.89	-1.84 SRPX	sushi-repeat containing protein, X-linked	Multiple_Complex
TC0300009	4.22	5.1	-1.84 VPS8	Transcript Identified by AceView, Entrez Gene ID(s) 23355	Unassigned
TC0900007	5.16	6.04	-1.84 SEMA4D	Memczak2013 ANTISENSE, coding, INTERNAL, UTR3 best transcri	NonCoding
TC1200010	5.45	6.33	-1.84 BCDIN3D	BCDIN3 domain containing	Multiple_Complex
TC1200012	7.5	8.38	-1.84 PRR4	proline rich 4 (lacrimal)	Multiple_Complex
TC0Y00006	4.83	5.71	-1.84 XKRY2; XKRY	Homo sapiens XK, Kell blood group complex subunit-related, Y-lin	Multiple_Complex
TC1100006	4.98	5.85	-1.84 PNPLA2	patatin-like phospholipase domain containing 2	Multiple_Complex
TC0200016	7.87	8.75	-1.84 CNNM3	cyclin and CBS domain divalent metal cation transport mediator 3	Multiple_Complex
TC1200012	5.5	6.38	-1.84 CLEC4A	C-type lectin domain family 4, member A	Coding
TC0100009	7.98	8.86	-1.84 SARS	seryl-tRNA synthetase	Multiple_Complex
TC0100008	4.09	4.96	-1.84 ADGRL2	adhesion G protein-coupled receptor L2	Multiple_Complex
TC1800008	4.38	5.26	-1.83 ANKRD29	ankyrin repeat domain 29	Multiple_Complex
TC1000011	5.92	6.8	-1.83 DLG5	discs, large homolog 5 (Drosophila)	Multiple_Complex
TC0800011	3.26	4.14	-1.83 FAM135B	family with sequence similarity 135, member B	Multiple_Complex
TC0200016	9.88	10.75	-1.83 MSH6	mutS homolog 6	Multiple_Complex
TC1700011	4.66	5.54	-1.83 CDC42EP4	CDC42 effector protein (Rho GTPase binding) 4	Multiple_Complex
TC1600011	9.08	9.95	-1.83 TERF2	telomeric repeat binding factor 2	Multiple_Complex
TC0500013	6.16	7.03	-1.83 MTRR	5-methyltetrahydrofolate-homocysteine methyltransferase reduc	NonCoding
TC0300012	5.3	6.17	-1.83 HCLS1	hematopoietic cell-specific Lyn substrate 1	Multiple_Complex
TC1600007	4.01	4.88	-1.83 ZG16	zymogen granule protein 16	Multiple_Complex
TC0700009	9.43	10.31	-1.83 CCDC136	coiled-coil domain containing 136	Multiple_Complex
TC0900009	5.71	6.58	-1.83 NOL6	nucleolar protein 6 (RNA-associated)	Multiple_Complex
TC1400010	5.25	6.12	-1.83 MDP1; NEDD8-MDP1; NEDD8; CHMP	magnesium-dependent phosphatase 1; NEDD8-MDP1 readthroug	Multiple_Complex
TC0900011	7.93	8.8	-1.83 OBP2B	odorant binding protein 2B	Multiple_Complex
TC1600008	9.56	10.43	-1.83 CENPN	centromere protein N	Multiple_Complex

TC0600008	7.6	8.47	-1.83	MYO6	myosin VI	Multiple_Complex
TC0600009	9.61	10.48	-1.83	SMPDL3A	sphingomyelin phosphodiesterase, acid-like 3A	Multiple_Complex
TC0400009	7.36	8.23	-1.83	ETFDH	electron-transferring-flavoprotein dehydrogenase	Multiple_Complex
TC1700011	3.5	4.36	-1.83	CYB561	cytochrome b561	Multiple_Complex
TC1100010	4.32	5.19	-1.83	APIP	Transcript Identified by AceView, Entrez Gene ID(s) 51074	Unassigned
TSUnmapp	4.82	5.69	-1.82	PRAMEF9		Coding
TC0200013	10	10.87	-1.82	BUB1	BUB1 mitotic checkpoint serine/threonine kinase	Multiple_Complex
TC0X00007	3.06	3.93	-1.82	NXF2B; NXF2	nuclear RNA export factor 2B; nuclear RNA export factor 2	Coding
TC1900008	8.71	9.57	-1.82	PLD3	phospholipase D family, member 3	Multiple_Complex
TC1800006	5.59	6.46	-1.82	LAMA3	laminin, alpha 3	Multiple_Complex
TC0800012	9	9.86	-1.82	DGAT1; MIR6848	diacylglycerol O-acyltransferase 1; microRNA 6848	Multiple_Complex
TC1000007	9.36	10.23	-1.82	MSRB2	methionine sulfoxide reductase B2	Multiple_Complex
TC0100013	6.87	7.73	-1.82	CLSPN	claspin	Multiple_Complex
TC1200009	4.17	5.03	-1.82	MANSC1	MANSC domain containing 1	Coding
TC0800011	3.03	3.89	-1.82	SAMD12	sterile alpha motif domain containing 12	Multiple_Complex
TC0100007	6.03	6.89	-1.82	SFN	stratifin	Coding
TC0400008	4.19	5.05	-1.82	MYOZ2	myozenin 2	Coding
TC0600007	6.04	6.9	-1.82	HIST1H3E	histone cluster 1, H3e	Coding
TC0300011	3.18	4.04	-1.82	ADAMTS9	ADAM metallopeptidase with thrombospondin type 1 motif 9	Multiple_Complex
TC0700010	3.26	4.12	-1.81	PRPS1L1	phosphoribosyl pyrophosphate synthetase 1-like 1	Coding
TC1200009	4.4	5.26	-1.81	CABP1	calcium binding protein 1	Multiple_Complex
TC2000008	6.38	7.24	-1.81	C20orf96	chromosome 20 open reading frame 96	Multiple_Complex
TC1700011	11.64	12.5	-1.81	SKA2	spindle and kinetochore associated complex subunit 2	Multiple_Complex
TC0200010	5.7	6.55	-1.81	CPS1	carbamoyl-phosphate synthase 1	Multiple_Complex
TC0500013	7.95	8.8	-1.81	PDCD6	programmed cell death 6	Multiple_Complex
TC1600008	3.96	4.81	-1.81	TSNAXIP1	translin-associated factor X interacting protein 1	Multiple_Complex
TC1800007	9.04	9.9	-1.81	SEC11C	SEC11 homolog C, signal peptidase complex subunit	Multiple_Complex
TC1200012	3.86	4.71	-1.81	GALNT8	polypeptide N-acetylgalactosaminyltransferase 8	Multiple_Complex
TC1700007	4.87	5.72	-1.81	MYO15A	myosin XVA	Multiple_Complex
TC0600011	3.58	4.43	-1.8	HLA-DOA	major histocompatibility complex, class II, DO alpha	Multiple_Complex
TC1700010	4.5	5.36	-1.8	KRT24	keratin 24, type I	Multiple_Complex
TC0100018	2.86	3.71	-1.8	CHRM3	cholinergic receptor, muscarinic 3	Multiple_Complex
TC1700010	6.17	7.02	-1.8	ATXN7L3	ataxin 7-like 3	Multiple_Complex
TC0700013	7.49	8.34	-1.8	MRPS24	mitochondrial ribosomal protein S24	Multiple_Complex
TC1600007	2.86	3.71	-1.8	IL21R	interleukin 21 receptor	Multiple_Complex
TC0400009	3.29	4.14	-1.8	CPE	carboxypeptidase E	Coding
TC1700010	4.85	5.7	-1.8	SLC47A2	solute carrier family 47 (multidrug and toxin extrusion), member	Multiple_Complex
TC0800012	7.83	8.68	-1.8	FNTA	farnesyltransferase, CAAX box, alpha	Multiple_Complex
TC1900011	4.08	3.08	2	SPINT2	serine peptidase inhibitor, Kunitz type, 2	Multiple_Complex
TC0300006	4.62	3.61	2	SYN2	synapsin II	Multiple_Complex
TC0500012	5.49	4.49	2	DRD1	dopamine receptor D1	Coding
TC0X00007	5	4	2	RPA4	replication protein A4	Coding
TC0700012	5.15	4.15	2	TRY2P	trypsinogen-like pseudogene	Multiple_Complex
TC1300008	10.68	9.68	2	GJB2	gap junction protein beta 2	Coding
TC0400009	6.48	5.48	2	MFSD7	major facilitator superfamily domain containing 7	Multiple_Complex
TC1200009	3.45	2.44	2.01	TAS2R8	taste receptor, type 2, member 8	Coding
TC0X00011	5.7	4.69	2.01	PNCK	pregnancy up-regulated nonubiquitous CaM kinase	NonCoding

TC1000008	5.23	4.22	2.01	LIPN	lipase, family member N	Coding
TC0200013	5.56	4.55	2.01	LYG2	lysozyme G-like 2	Coding
TC0400006	4.38	3.38	2.01	CD38	CD38 molecule	Multiple_Complex
TC0600013	5.2	4.19	2.01	TMEM244	transmembrane protein 244	Coding
TC0900010	6.21	5.2	2.01	GNA14	guanine nucleotide binding protein (G protein), alpha 14	Multiple_Complex
TC1100012	4.6	3.59	2.01	NXPE4	neurexophilin and PC-esterase domain family, member 4	Coding
TC0600006	6.56	5.55	2.01	GCNT6	glucosaminyl (N-acetyl) transferase 6 [Source:HGNC Symbol;Acc:	Pseudogene
TC1600009	5.12	4.11	2.01	FAM57B	family with sequence similarity 57, member B	Multiple_Complex
TC0300011	5.49	4.47	2.02	FEZF2	FEZ family zinc finger 2	Coding
TC0300007	6.99	5.98	2.02	MOBP	myelin-associated oligodendrocyte basic protein	Multiple_Complex
TC1700012	5.56	4.55	2.02	HOXB-AS3	HOXB cluster antisense RNA 3	Multiple_Complex
TC0500009	3.91	2.89	2.02	GRIA1	Transcript Identified by AceView, Entrez Gene ID(s) 2890	Unassigned
TC1900007	10.84	9.83	2.02	GAPDHS	glyceraldehyde-3-phosphate dehydrogenase, spermatogenic	Coding
TC0800006	6.39	5.38	2.02	SLC35G5	solute carrier family 35, member G5	Coding
TC0600007	4.26	3.25	2.02	NKAPL	NFKB activating protein-like	Coding
TC0600009	5.15	4.13	2.02	RNF217	ring finger protein 217	Multiple_Complex
TC0500009	4.84	3.82	2.02	SPINK9	serine peptidase inhibitor, Kazal type 9	Coding
TC0800008	4.71	3.69	2.02	RAD21-AS1	RAD21 antisense RNA 1	NonCoding
TC0300007	5.14	4.12	2.03	TMIE	transmembrane inner ear	Coding
TC2200008	5.35	4.33	2.03	DNAJB7	DnaJ (Hsp40) homolog, subfamily B, member 7	Coding
TC0300009	4.2	3.18	2.03	ARHGEF26	Rho guanine nucleotide exchange factor 26	Multiple_Complex
TC1900009	4.92	3.9	2.03	ODF3L2	outer dense fiber of sperm tails 3-like 2	Multiple_Complex
TC0100007	6.74	5.72	2.03	RCAN3	RCAN family member 3	Multiple_Complex
TC0200014	5.38	4.36	2.03	NEB	nebulin	Multiple_Complex
TC2100008	7.34	6.32	2.03	KRTAP12-1	keratin associated protein 12-1	Coding
TC1900008	8.91	7.89	2.03	AKT1S1	Memczak2013 ANTISENSE, coding, INTERNAL, intronic best transc	NonCoding
TSUnmapp	7.34	6.31	2.03	SLC16A1	solute carrier family 16 (monocarboxylate transporter), member	NonCoding
TC1200008	5.14	4.12	2.03	LRRIQ1	leucine-rich repeats and IQ motif containing 1	Multiple_Complex
TC1000011	8.69	7.67	2.03	LOXL4	lysyl oxidase-like 4	Multiple_Complex
TC1200012	10.93	9.91	2.03	SSPN	sarcospan	Multiple_Complex
TC0900007	4.99	3.97	2.03	SPATA31D4	SPATA31 subfamily D, member 4	Coding
TC0500013	4.39	3.37	2.03	SIL1	SIL1 nucleotide exchange factor	NonCoding
TC1100011	5.75	4.72	2.04	VSTM5	V-set and transmembrane domain containing 5	Multiple_Complex
TC0500012	7.99	6.96	2.04	FCHSD1	FCH and double SH3 domains 1	Multiple_Complex
TC2200008	7	5.98	2.04	SULT4A1	sulfotransferase family 4A member 1	Multiple_Complex
TC1700008	4.41	3.39	2.04	OR4D1	olfactory receptor, family 4, subfamily D, member 1	Coding
TC2000009	5.02	4	2.04	WFDC8	WAP four-disulfide core domain 8	Coding
TC0200014	8.65	7.62	2.04	BIN1	bridging integrator 1	Multiple_Complex
TC0100009	5.35	4.32	2.04	CHI3L2	chitinase 3-like 2	Multiple_Complex
TC1900011	7.41	6.38	2.04	FXYD1	FXYD domain containing ion transport regulator 1	Multiple_Complex
TC1700009	4.59	3.56	2.04	HP09025; CTD-2116F7.1	uncharacterized LOC100652929; novel transcript; uncharacterize	NonCoding
TC0800008	4.56	3.53	2.04	PKIA	protein kinase (cAMP-dependent, catalytic) inhibitor alpha	Multiple_Complex
TC0700012	4.73	3.7	2.04	TAS2R16	taste receptor, type 2, member 16	Coding
TC1100011	5.05	4.02	2.05	PYGM	phosphorylase, glycogen, muscle	Multiple_Complex
TC0300009	5.29	4.26	2.05	TM4SF1-AS1	TM4SF1 antisense RNA 1	Multiple_Complex
TC0700008	4.81	3.77	2.05	LMOD2	leiomodin 2 (cardiac)	Coding
TC1400007	4.37	3.34	2.05	KCNK13	potassium channel, two pore domain subfamily K, member 13	Coding

TC0100015	4.85	3.82	2.05 HORMAD1	HORMA domain containing 1	Multiple_Complex
TC0900009	4.68	3.65	2.05 GLIS3	GLIS family zinc finger 3	Multiple_Complex
TC1000011	9.35	8.32	2.05 ACTA2	actin, alpha 2, smooth muscle, aorta	Multiple_Complex
TC0500007	5.77	4.74	2.05 ITGA2	integrin, alpha 2 (CD49B, alpha 2 subunit of VLA-2 receptor)	Multiple_Complex
TC1600010	5.53	4.49	2.05 SALL1	spalt-like transcription factor 1	Multiple_Complex
TC0700011	7.8	6.76	2.05 C7orf43; MIR4658	chromosome 7 open reading frame 43; microRNA 4658	Multiple_Complex
TC0X00009	4.51	3.47	2.06 ARHGAP6	Rho GTPase activating protein 6	Multiple_Complex
TC0100007	5.53	4.48	2.06 VWA5B1	von Willebrand factor A domain containing 5B1	Multiple_Complex
TC1800007	5.33	4.28	2.07 ONECUT2	one cut homeobox 2	Multiple_Complex
TC0200012	7.28	6.24	2.07 SLC30A3	solute carrier family 30 (zinc transporter), member 3	Multiple_Complex
TC1700010	5.93	4.88	2.07 KIF18B	Memczak2013 ALT_ACCEPTOR, coding, INTERNAL, intronic best t	NonCoding
TC1400008	5.31	4.26	2.07 OR4K13	olfactory receptor, family 4, subfamily K, member 13	Coding
TC2200008	6.75	5.7	2.07 IGLL1	immunoglobulin lambda-like polypeptide 1	Coding
TC1000009	7.75	6.7	2.07 NANOS1	nanos homolog 1 (Drosophila)	Coding
TC1900011	4.64	3.59	2.07 ZNF846	zinc finger protein 846	NonCoding
TC1500010	5.89	4.83	2.07 GOLGA6C	golgin A6 family, member C	Coding
TC2000008	4.78	3.72	2.08 CST9L	cystatin 9-like	Coding
TC0500011	4.77	3.72	2.08 F2RL2	coagulation factor II (thrombin) receptor-like 2	Coding
TC0500009	4.86	3.8	2.08 NIPAL4	NIPA-like domain containing 4	Multiple_Complex
TC0900012	4.48	3.42	2.08 IFNA10	interferon, alpha 10	Coding
TC0800011	5.51	4.45	2.08 KCNV1	potassium channel, voltage gated modifier subfamily V, member	Multiple_Complex
TC1600011	8.29	7.23	2.08 WFIKKN1	WAP, follistatin/kazal, immunoglobulin, kunitz and netrin domain	Coding
TC1000011	7.45	6.4	2.08 MYOF	myoferlin	Multiple_Complex
TC0300013	10.09	9.04	2.08 CLDN1	claudin 1	Multiple_Complex
TC2100008	7.63	6.57	2.08 KRTAP12-2	keratin associated protein 12-2	Coding
TC2000007	5.1	4.04	2.08 SPINT4	serine peptidase inhibitor, Kunitz type 4	Coding
TC0600011	5.75	4.69	2.09 CYP39A1	cytochrome P450, family 39, subfamily A, polypeptide 1	Multiple_Complex
TC1300009	5.96	4.9	2.09 NALCN	sodium leak channel, non selective	Multiple_Complex
TC0900009	6.61	5.54	2.09 PAX5	paired box 5	Multiple_Complex
TC0200012	5.1	4.04	2.1 CGREF1	cell growth regulator with EF-hand domain 1	Multiple_Complex
TC1600011	5.7	4.63	2.1 ACSM3	acyl-CoA synthetase medium-chain family member 3	Multiple_Complex
TC1100012	6.63	5.56	2.1 IGSF9B	immunoglobulin superfamily, member 9B	Multiple_Complex
TC1100009	4.22	3.15	2.1 OR51G1	olfactory receptor, family 51, subfamily G, member 1 (gene/pseu	Multiple_Complex
TC1100009	4.92	3.84	2.1 PDZD3	PDZ domain containing 3	Multiple_Complex
TC0100006	4.39	3.32	2.1 PRAMEF20	PRAME family member 20	Coding
TC1700010	5.91	4.84	2.1 NUFIP2; rerdy	Memczak2013 ALT_ACCEPTOR, ALT_DONOR, coding, INTERNAL, i	Multiple_Complex
TC1700006	6.34	5.27	2.1 ENO3	enolase 3 (beta, muscle)	Multiple_Complex
TC1000011	5.86	4.78	2.11 NRAP	nebulin-related anchoring protein	Coding
TC1000008	4.75	3.68	2.11 LIPJ	lipase, family member J	Multiple_Complex
TC1600007	6.1	5.02	2.11 CHD9	Transcript Identified by AceView, Entrez Gene ID(s) 80205	Unassigned
TC0300006	5.44	4.36	2.11 SLC6A11	solute carrier family 6 (neurotransmitter transporter), member 1	Multiple_Complex
TC0100012	4.73	3.65	2.11 OR2L5	olfactory receptor, family 2, subfamily L, member 5	Coding
TC1100007	4.54	3.46	2.11 OR5A1	olfactory receptor, family 5, subfamily A, member 1	Coding
TC0500013	5.92	4.84	2.11 ACSL6	acyl-CoA synthetase long-chain family member 6	Multiple_Complex
TC0300011	6.77	5.69	2.11 FHIT	fragile histidine triad	Multiple_Complex
TC0800008	8.01	6.93	2.11 WWP1	Transcript Identified by AceView, Entrez Gene ID(s) 11059	Unassigned
TC1900011	7.05	5.97	2.11 RRAS	related RAS viral (r-ras) oncogene homolog	Multiple_Complex

TC2100007	4.83	3.74	2.12	GRIK1	glutamate receptor, ionotropic, kainate 1	Multiple_Complex
TC0200008	6.79	5.71	2.12	TLX2	T-cell leukemia homeobox 2	Multiple_Complex
TC0100016	4.13	3.04	2.12	CD84	CD84 molecule	Multiple_Complex
TC0300013	5.27	4.19	2.12	LAMP3	lysosomal-associated membrane protein 3	Multiple_Complex
TC0600010	4.35	3.26	2.13	PNLDC1	poly(A)-specific ribonuclease (PARN)-like domain containing 1	Multiple_Complex
TC1200012	6.03	4.94	2.13	C1RL	complement component 1, r subcomponent-like	Multiple_Complex
TC0800010	4.12	3.03	2.13	TRPA1	transient receptor potential cation channel, subfamily A, member	Multiple_Complex
TC1000010	10.06	8.97	2.13	SGMS1	sphingomyelin synthase 1	Multiple_Complex
TC1000011	4.65	3.55	2.14	LIPA	Transcript Identified by AceView, Entrez Gene ID(s) 3988	Unassigned
TC1000006	4.46	3.37	2.14	TMEM236	transmembrane protein 236	Coding
TC1900011	6.21	5.11	2.14	ZSCAN18	zinc finger and SCAN domain containing 18	Multiple_Complex
TC1000010	5.58	4.47	2.15	SLC16A9	solute carrier family 16, member 9	Multiple_Complex
TC1200007	7.02	5.92	2.15	KRT86	keratin 86, type II	Multiple_Complex
TC1100012	4.31	3.21	2.15	PLET1	placenta expressed transcript 1	Multiple_Complex
TC0600011	4.19	3.09	2.15	ADGRF5	adhesion G protein-coupled receptor F5	Multiple_Complex
TC1300008	5.32	4.21	2.15	SIAH3	siah E3 ubiquitin protein ligase family member 3	Coding
TC1000010	4.65	3.55	2.15	DNAJC12	DnaJ (Hsp40) homolog, subfamily C, member 12	Multiple_Complex
TC0200009	4.02	2.91	2.15	KYNU	kynureninase	Multiple_Complex
TC2000009	7.59	6.48	2.15	NEURL2	neuralized E3 ubiquitin protein ligase 2	Coding
TC0100018	11.23	10.12	2.16	ARHGEF2	Rho/Rac guanine nucleotide exchange factor 2	Multiple_Complex
TC1200007	6.47	5.37	2.16	PCED1B	PC-esterase domain containing 1B	Multiple_Complex
TC0200014	9.41	8.3	2.16	SLC25A12	solute carrier family 25 (aspartate/glutamate carrier), member 12	Multiple_Complex
TC0X00008	4.66	3.56	2.16	CT45A1	cancer/testis antigen family 45, member A1	Coding
TC0400011	5.61	4.5	2.16	ARSJ	arylsulfatase family, member J	Multiple_Complex
TC1900008	4.64	3.53	2.16	KIR2DS5; KIR3DL2; KIR2DL1; KIR3DL3	killer cell immunoglobulin-like receptor, two domains, short cyto	Multiple_Complex
TC0100018	5.94	4.83	2.16	OR11L1	olfactory receptor, family 11, subfamily L, member 1	Coding
TC2000009	4.48	3.37	2.16	CYP24A1	cytochrome P450, family 24, subfamily A, polypeptide 1	Multiple_Complex
TC0100018	5.76	4.65	2.16	AGT	angiotensinogen (serpin peptidase inhibitor, clade A, member 8)	Multiple_Complex
TC1900011	5.25	4.14	2.16	VSIG10L	V-set and immunoglobulin domain containing 10 like	Multiple_Complex
TC1600009	4.42	3.31	2.17	SOCS1	suppressor of cytokine signaling 1	Coding
TC1600011	6.82	5.7	2.17	MVP; PAGR1	major vault protein; PAXIP1 associated glutamate-rich protein 1	Multiple_Complex
TC2000007	6.34	5.22	2.17	WISP2	WNT1 inducible signaling pathway protein 2	Multiple_Complex
TC1300007	4.79	3.67	2.18	IRG1	immunoresponsive 1 homolog (mouse)	Coding
TC1200011	3.67	2.54	2.18	RASSF9	Ras association (RalGDS/AF-6) domain family (N-terminal) memb	Coding
TC1700010	5.47	4.35	2.18	CCL3	chemokine (C-C motif) ligand 3	Multiple_Complex
TC0600010	4.7	3.58	2.18	F13A1	coagulation factor XIII, A1 polypeptide	Multiple_Complex
TC1100011	5.38	4.26	2.18	WNT11	wingless-type MMTV integration site family, member 11	Multiple_Complex
TC0300014	5.17	4.04	2.18	ANKUB1	ankyrin repeat and ubiquitin domain containing 1	Multiple_Complex
TC0200015	5.46	4.33	2.18	DIRC3	disrupted in renal carcinoma 3	Multiple_Complex
TC1400008	4.37	3.24	2.19	RNASE12; RNASE11	ribonuclease, RNase A family, 12 (non-active); ribonuclease, RNas	Multiple_Complex
TC1600007	6.78	5.65	2.19	ABCC1	Jeck2013 ALT_ACCEPTOR, ALT_DONOR, coding, INTERNAL, intron	NonCoding
TC1100013	5.26	4.12	2.2	FLRT1	fibronectin leucine rich transmembrane protein 1	Coding
TC0800007	4.16	3.02	2.2	ADAM18	ADAM metallopeptidase domain 18	Multiple_Complex
TC0300013	4.38	3.24	2.2	SSUH2	ssu-2 homolog (C. elegans)	Multiple_Complex
TSUnmapp	5.68	4.54	2.21	PADI3	peptidyl arginine deiminase, type III	Coding
TC0200013	7.76	6.62	2.21	GCC2	Jeck2013 ANTISENSE, CDS, coding, INTERNAL, intronic, OVCODE,	NonCoding
TC0300008	6.15	5.01	2.21	SIDT1	SID1 transmembrane family, member 1	Multiple_Complex

TC1500007	6.22	5.07	2.21	ARID3B	AT rich interactive domain 3B (BRIGHT-like)	Multiple_Complex
TC0500007	4.36	3.21	2.21	PTGER4	prostaglandin E receptor 4 (subtype EP4)	Multiple_Complex
TC0300013	7.9	6.75	2.21	ACKR2	atypical chemokine receptor 2	Multiple_Complex
TC1100007	3.91	2.77	2.21	OR5T3	olfactory receptor, family 5, subfamily T, member 3	Coding
TC0400010	6.16	5.01	2.21	LCORL	Transcript Identified by AceView, Entrez Gene ID(s) 254251	Coding
TC0100014	5.65	4.5	2.22	WDR78	WD repeat domain 78	Multiple_Complex
TC1600010	6.22	5.07	2.23	ENKD1	enkurin domain containing 1	Multiple_Complex
TC0300007	5.19	4.03	2.23	CAMP	cathelicidin antimicrobial peptide	Coding
TC0300013	6.02	4.86	2.23	CLCN2	chloride channel, voltage-sensitive 2	Multiple_Complex
TC1900009	3.98	2.82	2.23	OR7G3	olfactory receptor, family 7, subfamily G, member 3	Coding
TC0700011	5.1	3.94	2.24	SEMA3E	sema domain, immunoglobulin domain (Ig), short basic domain, s	Coding
TC1700012	8.9	7.74	2.24	ITGB3	integrin beta 3	Multiple_Complex
TC1100009	4.68	3.51	2.24	OR56A5	olfactory receptor, family 56, subfamily A, member 5	Coding
TC1200009	7.49	6.32	2.24	YBX3	Y box binding protein 3	Multiple_Complex
TC0200007	7.06	5.89	2.25	LTBP1	latent transforming growth factor beta binding protein 1	Multiple_Complex
TC0900012	6.48	5.31	2.25	ALDH1A1	aldehyde dehydrogenase 1 family, member A1	Multiple_Complex
TC0X00009	4.43	3.26	2.25	SLC38A5	solute carrier family 38, member 5	Multiple_Complex
TC1100010	7.03	5.85	2.26	CELF1	Memczak2013 ALT_ACCEPTOR, ALT_DONOR, coding, INTERNAL, i	NonCoding
TC1300006	5.28	4.11	2.26	KL	klotho	Multiple_Complex
TC0600013	5.94	4.76	2.26	SYNE1	Memczak2013 ALT_ACCEPTOR, ALT_DONOR, coding, INTERNAL, i	NonCoding
TC0900009	4.64	3.46	2.26	OR13C7	olfactory receptor, family 13, subfamily C, member 7 (gene/pseu	Pseudogene
TC1200010	8.82	7.64	2.26	RARG	retinoic acid receptor, gamma	Multiple_Complex
TC0400011	4.85	3.67	2.26	INPP4B	inositol polyphosphate-4-phosphatase type II B	Multiple_Complex
TC1200007	6.25	5.07	2.27	NCKAP1L	NCK-associated protein 1-like	Multiple_Complex
TC0700009	4.95	3.77	2.27	GALNTL5	polypeptide N-acetylgalactosaminyltransferase-like 5	Multiple_Complex
TC0900012	8.71	7.53	2.27	GLE1	GLE1 RNA export mediator	Multiple_Complex
TC2200008	6.2	5.02	2.27	PVALB	parvalbumin	Multiple_Complex
TC1600006	4.53	3.35	2.27	A2BP1	Transcript Identified by AceView, Entrez Gene ID(s) 54715	Unassigned
TC0500007	5.07	3.88	2.28	IL7R	interleukin 7 receptor	Multiple_Complex
TSUnmapp	4.31	3.12	2.28	SAG	S-antigen; retina and pineal gland (arrestin)	Coding
TC1100012	5.02	3.83	2.28	MIR670HG; HSD17B12	MIR670 host gene; hydroxysteroid (17-beta) dehydrogenase 12	Multiple_Complex
TC1900011	6.53	5.34	2.28	ZBTB45	Memczak2013 ALT_ACCEPTOR, ALT_DONOR, coding, INTERNAL, i	NonCoding
TC1800007	4.89	3.7	2.29	SETBP1	Memczak2013 ALT_ACCEPTOR, ALT_DONOR, coding, INTERNAL, i	NonCoding
TC1100007	6.3	5.1	2.29	CD5	CD5 molecule	Multiple_Complex
TC0300008	6.37	5.17	2.29	ALDH1L1-AS1	ALDH1L1 antisense RNA 1	Multiple_Complex
TC0700008	9.69	8.49	2.29	LRRN3	leucine rich repeat neuronal 3	Multiple_Complex
TSUnmapp	4.57	3.38	2.29	SAG	S-antigen; retina and pineal gland (arrestin)	Coding
TC0X00006	5.89	4.7	2.29	MAGEB10	MAGE family member B10	Coding
TC1200011	4.43	3.23	2.3	MYL2	myosin light chain 2	Multiple_Complex
TC0200014	5.92	4.72	2.3	FAP	fibroblast activation protein alpha	Multiple_Complex
TC1000009	4.45	3.24	2.3	EMX2	empty spiracles homeobox 2	Multiple_Complex
TC0300012	4.09	2.89	2.3	CD200R1L	CD200 receptor 1 like	Coding
TC1800007	4.33	3.12	2.3	SERPINB11	serpin peptidase inhibitor, clade B (ovalbumin), member 11 (gene	Multiple_Complex
TC0300010	7.8	6.59	2.31	PRSS42	protease, serine 42	Coding
TC0500012	5.62	4.41	2.31	SAR1B	Transcript Identified by AceView, Entrez Gene ID(s) 51128	Unassigned
TC1100013	5.74	4.54	2.31	TEX40	testis expressed 40	Multiple_Complex
TC0200009	6.35	5.14	2.31	GPR17	G protein-coupled receptor 17	Multiple_Complex

TC0500011	7.07	5.86	2.31 LNPEP	Memczak2013 ANTISENSE, coding, INTERNAL, UTR3 best transcri	NonCoding
TC1100011	4.05	2.84	2.31 DLG2	discs, large homolog 2 (Drosophila)	Multiple_Complex
TC1000010	4.14	2.93	2.31 ZNF488	zinc finger protein 488	Coding
TC1100007	5.72	4.51	2.32 OOSP2	oocyte secreted protein 2	Multiple_Complex
TC0700013	4	2.78	2.32 MACC1	metastasis associated in colon cancer 1	Multiple_Complex
TC0100016	4.79	3.58	2.32 FCGR3A	Fc fragment of IgG, low affinity IIIa, receptor (CD16a)	Multiple_Complex
TC2000009	5.02	3.8	2.32 FAM209B	family with sequence similarity 209, member B	Coding
TC1200011	5.06	3.84	2.32 CMKLR1	chemerin chemokine-like receptor 1	Multiple_Complex
TC0100011	6.6	5.38	2.33 SERTAD4	SERTA domain containing 4	Multiple_Complex
TC0600011	4.69	3.47	2.33 OR12D3	olfactory receptor, family 12, subfamily D, member 3	Coding
TC1700009	6.44	5.22	2.33 MYH2	myosin, heavy chain 2, skeletal muscle, adult	Multiple_Complex
TC0400007	5.46	4.24	2.33 HTN3	histatin 3	Multiple_Complex
TC0600012	4.59	3.36	2.33 CRISP2	cysteine-rich secretory protein 2	Coding
TC0500012	5.76	4.54	2.34 SPOCK1	sparc/osteonectin, cwcv and kazal-like domains proteoglycan (tes	Multiple_Complex
TC1000008	10.11	8.88	2.34 RP11-380G5.2; PTEN	Jeck2013 ALT_ACCEPTOR, ALT_DONOR, coding, INTERNAL, intron	NonCoding
TC0400006	4.91	3.68	2.34 ZNF141	Transcript Identified by AceView, Entrez Gene ID(s) 100288237; 7	Coding
TC0500012	4.55	3.32	2.34 SLC25A2	solute carrier family 25 (mitochondrial carrier; ornithine transpor	Coding
TC0100009	8.37	7.14	2.34 LRIG2	leucine-rich repeats and immunoglobulin-like domains 2	Multiple_Complex
TC0200011	4.09	2.86	2.34 SPATA3	spermatogenesis associated 3	Multiple_Complex
TC1200010	6.21	4.98	2.34 ZNF385A	zinc finger protein 385A	Multiple_Complex
TSUnmapp	4.57	3.34	2.35 SAG	S-antigen; retina and pineal gland (arrestin)	NonCoding
TC0400006	4.88	3.64	2.35 USP17L12	ubiquitin specific peptidase 17-like family member 12	Coding
TC0800009	4.91	3.68	2.36 DENND3	Memczak2013 ALT_ACCEPTOR, ALT_DONOR, coding, INTERNAL, i	NonCoding
TC1700008	6.51	5.27	2.36 SGCA	sarcoglycan alpha	Multiple_Complex
TC1100006	6.73	5.5	2.36 OR52N2	olfactory receptor, family 52, subfamily N, member 2	Coding
TC0100013	4.62	3.38	2.36 ADGRB2	adhesion G protein-coupled receptor B2	Multiple_Complex
TC1500010	4.29	3.05	2.36 CTXN2	cortexin 2	Coding
TC0300011	4.28	3.04	2.37 ASB14	ankyrin repeat and SOCS box containing 14	Multiple_Complex
TC0Y00006	4.98	3.74	2.37 SRY	sex determining region Y	Coding
TC1600007	7.78	6.53	2.37 ATXN2L	Transcript Identified by AceView, Entrez Gene ID(s) 11273	Unassigned
TC1000008	7.49	6.24	2.37 IFIT2	interferon-induced protein with tetratricopeptide repeats 2	Coding
TC1000007	5	3.75	2.37 PTPN20	protein tyrosine phosphatase, non-receptor type 20	Multiple_Complex
TC1900007	4.35	3.11	2.38 NANOS3	nanos homolog 3 (Drosophila)	Multiple_Complex
TC0800006	7.12	5.87	2.38 BLK	Transcript Identified by AceView, Entrez Gene ID(s) 640	Unassigned
TC1100010	4.89	3.64	2.38 SLC5A12	solute carrier family 5 (sodium/monocarboxylate cotransporter),	Multiple_Complex
TC0500012	5.15	3.9	2.38 SPINK1	serine peptidase inhibitor, Kazal type 1	Multiple_Complex
TC0800007	5.12	3.86	2.39 IDO1	indoleamine 2,3-dioxygenase 1	Multiple_Complex
TC0300007	5.37	4.11	2.39 TOPAZ1	testis and ovary specific PAZ domain containing 1	Coding
TC1300008	7.13	5.87	2.39 TUBGCP3	Memczak2013 ANTISENSE, coding, INTERNAL, intronic best transc	NonCoding
TC0200015	4.72	3.46	2.4 ERBB4	Transcript Identified by AceView, Entrez Gene ID(s) 2066	Unassigned
TC1700010	9.94	8.68	2.4 KRT17	keratin 17, type I	Multiple_Complex
TC1700009	5.18	3.92	2.4 RCVRN	recoverin	Multiple_Complex
TC0600013	7.33	6.06	2.41 HIVEP2	Jeck2013 ALT_ACCEPTOR, ALT_DONOR, coding, INTERNAL, intron	NonCoding
TC0800011	6.85	5.58	2.41 ABRA	actin binding Rho activating protein	Coding
TC1500010	5.27	3.99	2.43 POLR2M	polymerase (RNA) II (DNA directed) polypeptide M	NonCoding
TC1200007	7.41	6.13	2.44 HOXC8	homeobox C8	Multiple_Complex
TC0600009	5.43	4.15	2.44 FAM26E	family with sequence similarity 26, member E	Coding

TC1400008	5.34	4.05	2.44	PPP4R4	protein phosphatase 4, regulatory subunit 4	Multiple_Complex
TC1900007	6.72	5.44	2.44	TMEM38A	Transcript Identified by AceView, Entrez Gene ID(s) 79041	Unassigned
TC1100006	5.07	3.78	2.44	OR10A5	olfactory receptor, family 10, subfamily A, member 5	Coding
TC1900010	5.77	4.48	2.45	EXOC3L2	exocyst complex component 3-like 2	Coding
TC0500008	4.64	3.34	2.45	PKD2L2	polycystic kidney disease 2-like 2	Coding
TC0400007	4.9	3.6	2.45	TMPRSS11E	transmembrane protease, serine 11E	Coding
TC1900007	6.15	4.86	2.46	LOC101927572; AC002116.7; CLIP3	uncharacterized LOC101927572; Memczak2013 ANTISENSE, CDS,	Multiple_Complex
TC0500006	5.85	4.56	2.46	PLEKHG4B	Zhang2013 ALT_ACCEPTOR, ALT_DONOR, coding, INTERNAL, intr	NonCoding
TC1100010	5.85	4.55	2.46	CCDC179	coiled-coil domain containing 179	Multiple_Complex
TC1300007	5.1	3.8	2.46	DACH1	Memczak2013 ANTISENSE, coding, INTERNAL, intronic best transc	NonCoding
TC1900011	6.84	5.54	2.46	FUT5	fucosyltransferase 5 (alpha (1,3) fucosyltransferase)	Coding
TC1700010	7.07	5.76	2.47	KAT2A	Memczak2013 ALT_DONOR, coding, INTERNAL, intronic best tran	NonCoding
TC1900007	6.06	4.74	2.49	OR10H3	olfactory receptor, family 10, subfamily H, member 3	Coding
TC1500007	6.75	5.44	2.49	MYO9A	Memczak2013 ANTISENSE, CDS, coding, INTERNAL best transcript	NonCoding
TC1700008	4.89	3.58	2.49	CACNG5	calcium channel, voltage-dependent, gamma subunit 5	Coding
TC0700011	4.58	3.26	2.49	SEMA3A	Transcript Identified by AceView, Entrez Gene ID(s) 10371	Unassigned
TC1600011	5.99	4.67	2.49	GAS8-AS1	GAS8 antisense RNA 1	Multiple_Complex
TC1700011	5.75	4.43	2.5	ABCA6	ATP binding cassette subfamily A member 6	Multiple_Complex
TC0300011	4.53	3.21	2.5	GABRR3	gamma-aminobutyric acid (GABA) A receptor, rho 3 (gene/pseud	Multiple_Complex
TC1100007	4.67	3.35	2.5	OR5D13	olfactory receptor, family 5, subfamily D, member 13 (gene/pseu	Multiple_Complex
TC0300008	6.29	4.97	2.5	STXBP5L	syntaxin binding protein 5-like	Multiple_Complex
TC0100013	6.22	4.9	2.51	YARS	Transcript Identified by AceView, Entrez Gene ID(s) 8565	Unassigned
TC1700012	4.92	3.59	2.51	OMG	oligodendrocyte myelin glycoprotein	Multiple_Complex
TC0900007	6.35	5.02	2.51	PRSS3	protease, serine, 3	Multiple_Complex
TC0700011	4.76	3.43	2.51	HGF	Transcript Identified by AceView, Entrez Gene ID(s) 3082	Unassigned
TC1000007	6.1	4.77	2.52	ZNF32-AS3	ZNF32 antisense RNA 3	NonCoding
TC0100018	8.94	7.61	2.52	SMYD3	Jeck2013 ALT_ACCEPTOR, ALT_DONOR, coding, INTERNAL, intron	NonCoding
TC0200008	8.55	7.21	2.52	VAMP8	vesicle associated membrane protein 8	Multiple_Complex
TC1600008	9.14	7.8	2.53	RHBDF1	rhomboid 5 homolog 1 (Drosophila)	Multiple_Complex
TC0300011	4.66	3.31	2.53	MORC1	MORC family CW-type zinc finger 1	Coding
TC0100016	9.67	8.32	2.54	FAM129A	family with sequence similarity 129, member A	Multiple_Complex
TC0600014	4.17	2.81	2.56	C6orf229	chromosome 6 open reading frame 229	Multiple_Complex
TC0200015	5.63	4.27	2.57	C2orf83	chromosome 2 open reading frame 83	Multiple_Complex
TC1700006	5.79	4.43	2.57	SLC35G6	solute carrier family 35, member G6	Coding
TC1200009	4.67	3.31	2.57	STYK1	serine/threonine/tyrosine kinase 1	Coding
TC0100015	4.28	2.9	2.6	TBX15	T-box 15	Multiple_Complex
TC0800009	11.71	10.33	2.6	PTP4A3	protein tyrosine phosphatase type IVA, member 3	Multiple_Complex
TC1700007	4.85	3.47	2.61	GRAPL	GRB2-related adaptor protein-like	Coding
TC0200015	6.89	5.51	2.62	MYL1	myosin light chain 1	Multiple_Complex
TC0200016	7.14	5.74	2.64	ANKRD23	ankyrin repeat domain 23	Multiple_Complex
TC1400006	4.85	3.45	2.64	OR11G2	olfactory receptor, family 11, subfamily G, member 2	Coding
TC1500010	4.61	3.2	2.66	RPL4	ribosomal protein L4	NonCoding
TC0800011	5.67	4.26	2.66	KCNK9	potassium channel, two pore domain subfamily K, member 9	Multiple_Complex
TC0800009	5.35	3.94	2.67	DEFB105A	defensin, beta 105A	Multiple_Complex
TC0100011	5.3	3.88	2.68	C1orf106	chromosome 1 open reading frame 106	Multiple_Complex
TC0100016	4.57	3.14	2.69	KCNT2	potassium channel, sodium activated subfamily T, member 2	Multiple_Complex
TC1900007	5.03	3.6	2.69	WDR88	WD repeat domain 88	Coding

TC1200011	6.07	4.64	2.7	PTPRB	protein tyrosine phosphatase, receptor type, B	Multiple_Complex
TC0200007	5.76	4.33	2.7	KRTCAP3	keratinocyte associated protein 3	Multiple_Complex
TC0300012	6.61	5.18	2.7	PLSCR4	phospholipid scramblase 4	Multiple_Complex
TC0700007	6.94	5.5	2.72	WBSCR17	Memczak2013 ALT_ACCEPTOR, ALT_DONOR, coding, INTERNAL, i	NonCoding
TC2200007	7.02	5.58	2.72	FAM83F	family with sequence similarity 83, member F	Multiple_Complex
TC1000011	4.36	2.92	2.73	ANKRD1	ankyrin repeat domain 1 (cardiac muscle)	Multiple_Complex
TC1500007	5.58	4.13	2.73	CRABP1	cellular retinoic acid binding protein 1	Multiple_Complex
TC0700011	4.81	3.36	2.74	CYP3A7; CYP3A7-CYP3A51P	cytochrome P450, family 3, subfamily A, polypeptide 7; CYP3A7-C	Multiple_Complex
TC1400006	4.55	3.09	2.74	OR11H7	olfactory receptor, family 11, subfamily H, member 7 (gene/pseu	Pseudogene
TC0300008	5.02	3.56	2.74	GP9	glycoprotein IX (platelet)	Coding
TC1000009	5.13	3.67	2.74	PRKCQ	protein kinase C, theta	Multiple_Complex
TC0500012	5.24	3.79	2.74	C5orf46	chromosome 5 open reading frame 46	Multiple_Complex
TC1200010	4.7	3.23	2.76	FAM186A	family with sequence similarity 186, member A	Coding
TC1100012	4.89	3.42	2.76	OR8B3	olfactory receptor, family 8, subfamily B, member 3	Coding
TC1700009	6.15	4.64	2.85	MYH4	myosin, heavy chain 4, skeletal muscle	Coding
TC1000009	5.11	3.57	2.91	SKIDA1	SKI/DACH domain containing 1	Multiple_Complex
TC2000009	4.79	3.25	2.92	EMILIN3	elastin microfibril interfacer 3	Coding
TC0600011	5.75	4.2	2.92	OR2B3	olfactory receptor, family 2, subfamily B, member 3	Coding
TC2200007	7.64	6.09	2.94	CECR5	cat eye syndrome chromosome region, candidate 5	Multiple_Complex
TC1900008	5.31	3.73	2.98	PPP1R12C	Memczak2013 ANTISENSE, CDS, coding, INTERNAL best transcript	NonCoding
TC0600012	6.02	4.44	2.99	ASCC3	Memczak2013 ALT_ACCEPTOR, ALT_DONOR, coding, INTERNAL, i	NonCoding
TC0400011	6.13	4.54	3	PABPC4L	poly(A) binding protein, cytoplasmic 4-like	Coding
TC0400010	4.68	3.08	3.03	UGT2A3	UDP glucuronosyltransferase 2 family, polypeptide A3	Multiple_Complex
TC0100011	8.14	6.53	3.04	CD46	Transcript Identified by AceView, Entrez Gene ID(s) 4179	Unassigned
TC1400007	4.99	3.37	3.07	TSHR	thyroid stimulating hormone receptor	Multiple_Complex
TC0700006	6.12	4.49	3.1	UNCX	UNC homeobox	Coding
TC0X00008	7.54	5.89	3.14	FLNA	Jeck2013 ANTISENSE, CDS, coding, INTERNAL, OVCODE, OVEXON	NonCoding
TC1200008	4.85	3.19	3.16	MYBPC1	myosin binding protein C, slow type	Multiple_Complex
TC1200011	7.39	5.72	3.17	TMCC3; MIR7844	transmembrane and coiled-coil domain family 3; microRNA 7844	Multiple_Complex
TC0800011	4.37	2.7	3.18	SLC7A13	solute carrier family 7 (anionic amino acid transporter), member	Multiple_Complex
TC1700007	5.76	4.09	3.18	CTB-75G16.3; MRM1	Transcript Identified by AceView, Entrez Gene ID(s) 79922; novel	Multiple_Complex
TC0900009	9.24	7.54	3.25	TPM2	tropomyosin 2 (beta)	Multiple_Complex
TC1600009	5.39	3.69	3.25	SRL	sarcalumenin	Coding
TC0500007	4.6	2.88	3.28	CMYA5	cardiomyopathy associated 5	Multiple_Complex
TC2000007	5.72	4	3.3	DEFB115	defensin, beta 115	Coding
TC2100007	4.4	2.68	3.3	LOC388813; bafor; AF165138.7	uncharacterized protein ENSP00000383407-like; Transcript Identi	Multiple_Complex
TC1100008	5.31	3.55	3.39	IZUMO1R	IZUMO1 receptor, JUNO	Coding
TC1900007	6.16	4.36	3.48	KIAA1683	Memczak2013 ANTISENSE, CDS, coding, INTERNAL best transcript	NonCoding
TC0600012	5.25	3.43	3.53	KLHL31	kelch-like family member 31	Coding
TC1900008	8.42	6.58	3.57	RYR1	ryanodine receptor 1 (skeletal)	Multiple_Complex
TC1600007	8.29	6.45	3.59	MYLPF	myosin light chain, phosphorylatable, fast skeletal muscle	Multiple_Complex
TC0400009	6.79	4.92	3.64	1-Mar	Memczak2013 ANTISENSE, coding, INTERNAL, intronic best transc	NonCoding
TC0200015	8.69	6.82	3.65	TUBA4A	tubulin, alpha 4a	Multiple_Complex
TC0900008	6.38	4.49	3.72	TLR4	toll-like receptor 4	Multiple_Complex
TC0800010	5.78	3.88	3.74	IL7	interleukin 7	Multiple_Complex
TC1200012	6.72	4.82	3.75	POLE	Transcript Identified by AceView, Entrez Gene ID(s) 5426	Unassigned
TC1200012	8.03	6.11	3.76	RDH5	retinol dehydrogenase 5 (11-cis/9-cis)	Multiple_Complex

TC2000009	9.19	7.27	3.8	TNNC2	troponin C type 2 (fast)	Coding
TC1000012	4.63	2.66	3.93	LIPA	lipase A, lysosomal acid, cholesterol esterase	NonCoding
TC2200008	7.96	5.85	4.31	MB	myoglobin	Multiple_Complex
TC1700009	7.06	4.84	4.64	MYH1	myosin, heavy chain 1, skeletal muscle, adult	Coding
TC0200010	7.84	5.44	5.29	DES	desmin	Multiple_Complex
TC0400012	5.72	3.27	5.48	SMR3A	submaxillary gland androgen regulated protein 3A	Coding
TC2100006	4.91	2.24	6.38	NCAM2	Salzman2013 ALT_ACCEPTOR, ALT_DONOR, coding, INTERNAL, in	NonCoding
TC1900010	8.68	5.45	9.35	CKM	creatine kinase, muscle	Multiple_Complex
TC1600007	9.74	6.04	12.98	ATP2A1	ATPase, Ca++ transporting, cardiac muscle, fast twitch 1	Multiple_Complex
TC1100006	9.38	5.3	16.9	TNNT3	troponin T type 3 (skeletal, fast)	Multiple_Complex
